# What Are the Effects of Self-Regulation Phases and Strategies for Chinese Students? A Meta-Analysis of Two Decades Research of the Association Between Self-Regulation and Academic Performance

**DOI:** 10.3389/fpsyg.2018.02434

**Published:** 2018-12-18

**Authors:** Junyi Li, Hui Ye, Yun Tang, Zongkui Zhou, Xiangen Hu

**Affiliations:** ^1^School of Teacher Education and Psychology, Sichuan Normal University, Chengdu, China; ^2^School of Psychology, Central China Normal University, Wuhan, China; ^3^Central China Normal University Branch, Collaborative Innovation Center of Assessment toward Basic Education Quality, Wuhan, China; ^4^Key Laboratory of Adolescent Cyberpsychology and Behavior, Ministry of Education, Wuhan, China; ^5^Institute for Intelligent Systems, University of Memphis, Memphis, TN, United States

**Keywords:** self-regulated learning, academic achievement, elementary and secondary education, cross-sectional studies, meta-analysis

## Abstract

**Background:** Self-regulated learning refers to the monitoring and controlling of one's own cognitive performance before, during, and after a learning episode. Previous literature suggested that self-regulated learning had a significant relationship with academic achievement, but not all self-regulated learning strategies exerted the same influences. Using an invalid strategy may waste the limited psychological resources, which will cause the ego depletion effect. The present meta-analysis study intended to search for the best self-regulated learning strategies and inefficient strategies for Chinese students in elementary and secondary school, and analyzed the critical phases of self-regulated learning according to Zimmerman's theory. The moderating effects of gender, grade, and publication year were also analyzed.

**Methods:** Empirical studies which conducted in real teaching situations of elementary and secondary education were systematically searched using Chinese academic databases. Studies focused on undergraduate students, students of special education, or online learning environments were excluded. Fifty-five cross-sectional studies and four intervention studies (which generated 264 independent samples) were included with a total sample size of 23,497 participants. Random effects model was chosen in the current meta-analysis, and publication bias was also examined.

**Results:** The results indicated that the overall effect size of self-regulated learning on academic achievement was small for primary and secondary school students in China. The effect sizes of self-efficacy, task strategies, and self-evaluation were relatively higher than other strategies. Self-regulated learning strategies have the largest effect size on science disciplines (including mathematics and physics). Performance phase and self-reflection phase are key phases of self-regulated learning. From 1998 to 2016, the effect size between self-regulated learning and academic achievement was gradually decreasing.

**Conclusions:** The main findings of the current study showed that self-efficacy, task strategies, and self-evaluation were key self-regulated learning strategies for Chinese students. Performance phase and self-reflection phase played significant roles in the process of self-regulated learning. Future studies need to include more intervention studies with rigorous treatment fidelity control and provide more empirical evidence from online learning, so as to compare the different effects of self-regulated learning between traditional education and online education.

## Introduction

“How can one become a professional learner?” is one of the most important topics in educational psychology. Professional learners are good at using strategies to help their study and holding openness view to difficult tasks, and they are willing to accept challenges until they reach the goal. Using effective learning strategies, having strong self-efficacy and will power are significant characteristics of professional learners (Sternberg and Williams, 2010), all of which are characteristics of self-regulated learning (SRL). Unlike measures of mental ability or academic performance skill, self-regulated learning refers to the self-directive processes and self-beliefs that enable learners to transform their mental abilities into an academic performance skill (Zimmerman, 2008). Self-regulated learning also has been highly praised as the key competence to initiate and maintain lifelong learning (EU Council, 2002). Self-regulated learning could have a wide influence in areas, including subjective well-being, physical health, social achievement, economy, and online education (Mischel et al., 2010; Kizilcec et al., 2017). In education, researchers have found that self-regulated learning has a significant association with performance self-efficacy, learning motivation, and conscientiousness (Pintrich, 2004; Fernandez-Rio et al., 2017). These important traits also promote academic performance (Everaert et al., 2017; Street et al., 2017; Pascoe et al., 2018). Thus, the relationship between self-regulated learning and academic performance have attracted much attention.

## Self-Regulated Learning Strategies and Academic Performance

In Western countries, researchers have proved the effectiveness of self-regulated learning on academic achievement (Paris and Paris, 2001; Dignath et al., 2008; Sadati and Simin, 2017) and learning motivation (Pintrich, 1999). In China, Liu and Chen (2000) found that online self-regulated learning positively predicted writing scores of undergraduate students. Li et al. (2015) suggested that time value, time monitor, and self-efficacy have significant association with academic achievement in elementary school students. In secondary school, the effectiveness of self-regulated learning on academic achievement has also been verified (Chen and Hu, 2008; Zhang et al., 2012a). Therefore, there is a similar effect to Western education on the relationship between self-regulated learning and academic performance.

Self-regulated learning consists of many strategies, including goal setting, self-efficacy, goal orientation, metacognitive monitoring, self-evaluation and so on (Panadero, 2017). As an integrated conception, self-regulation promotes academic performance, but meta-analysis study indicated that not all self-regulated strategies are effective. Specifically, rehearsal learning, organization, and peer learning were found not significantly associated with GPA (Richardson et al., 2012). To explain the lack of effectiveness, Baumeister et al. (1994) proposed the strength model of self-control which suggested that psychological resource could be consumed by using self-regulated strategies. If the preceding self-regulated strategy consumes too much psychological resource, the performance of the subsequent strategy would decrease, leading to ego depletion (Li et al., 2011a). These results indicated that it is necessary to find out the most effective strategies for different students or teaching environments, so that we can save psychological resources and promote academic performance to the greatest extent. Thus, the first goal of the present study was to identify the best self-regulated learning strategy and invalid strategy for Chinese students in primary and secondary school.

## Phases and Sub-Processes of Self-Regulation

Self-regulated learning is a multidimensional construct that emphasizes the active role of the learner (Panadero, 2017). There are many definitions of self-regulation, and scholars did not reach a consensus in different area, even within educational psychology (Li et al., 2011a; Zimmerman, 2008). For example, the distinction between self-regulated learning and metacognitive learning strategies is often a fuzzy one that lacks clarification (Dignath et al., 2008). Metacognition and self-regulation sometimes refer to the same concept, whereas other models view metacognitive strategies as an important element of self-regulation. The terminology of self-regulation is also confounding, referred to as self-control, self-management, or metacognition in different areas. Hence, a comprehensive theory for SRL is needed to settle this problem. In the present study, we turned to the cyclical model of SRL proposed by Zimmerman (2008). It assumes bidirectional relationships of SRL processes across three phases, namely, forethought phase, performance phase (online SRL), and self-reflection phase (offline SRL). There is growing empirical evidences for this model: studies have indicated that students' self-efficacy was positively correlated with academic achievement (Chen, 2011; Doménechbetoret et al., 2017; Street et al., 2017). Microanalyses of self-regulated processes and sources of motivation have been used most frequently to investigate learning of athletic skills, such as free-throw shooting, volleyball serving and dart throwing, and these measures of SRL revealed significant differences between experts, non-experts, and novices (Zimmerman, 2008; Cleary and Zimmerman, 2010). When compared to non-experts and novices, experts made the most extensive use of SRL processes and reported the most positive motivational beliefs and feelings (Kitsantas and Zimmerman, 2002). Furthermore, research showed that novices who were taught multiple strategies displayed significantly greater athletic skill and improved motivational beliefs during relatively brief practice sessions than novices in a control group (Cleary et al., 2006). These evidences indicated that Zimmerman's model could distinguish between novices and experts effectively, it also made a good synthesis and elaboration of otherwise confusing SRL strategies, which has been supported by relevant empirical studies. Consequently, the second goal of the present study was to explore the effect sizes of sub-processes of self-regulation (i.e., forethought phase, performance phase, and self-reflection phase) according to Zimmerman's model in Chinese school settings, so as to find the detailed relationship of each phase on academic achievement.

## Potential Moderators Between SRL and Academic Achievement

Most studies on SRL in school has been conducted with older students, because younger students in elementary school probably have difficulties with SRL (Paris and Newman, 1990; Zimmerman, 1990; Panadero, 2017). Take students' views of their own abilities and motivation as an example. Young children sometimes focus on the wrong criteria of ability and exaggerate the available evidence. They often confuse perceptions of academic ability with appropriate social behavior (Stipek and Tannatt, 1984). For example, researchers found that first-grade students believe that sharing distinguishes average and smart students and that children who receive a great deal of criticism are less able than their peers (Blumenfeld et al., 1982). Studies also revealed that compared with 11–12 years old students, 7–8 years olds relatively less reflected on their performance and seldom evaluated and controlled their cognitive abilities (Paris and Newman, 1990). Although some studies showed that preschool children already started to use SRL strategies, and young children do engage in activities to self-regulate their learning (Perry et al., 2002, 2004; Schneider and Lockl, 2002), the level of SRL of younger students could be different from that of older students.

In addition, there is empirical evidence illustrate that girls outperform boys in adjusting to school (Ortiz and Bornacelly, 2017), whereas the gender difference in students' self-regulation competence may be one of the important factors for girls' advantage (Jiao and Gai, 2011). Girls' self-control is significantly higher than that of boys at 2, 4, and 11 years old (Zhang et al., 2012b). A meta-analytic study about children's performance at the task of resisting the temptation have found that the performance of girls was better than boys (Silverman, 2002). From the insight of academic achievement, the motivations and emotions of girls and boys toward mathematics were probably significantly different. In K-12 education, girls reported increasingly more negative attitudes toward mathematics performance and showed more self-derogating attributions about their mathematics performance (Royer and Walles, 2007; Hyde et al., 2008). In early adolescence, gender differences have also been found for mathematics self-concept and mathematics utility (Eccles et al., 1993). These evidences suggested that the relationship between SRL and academic achievement may be moderated by gender.

Finally, the year of publication is an interesting variable, considering the rapid growth of economy and technology in China from 1990s to 2010s. To our knowledge, few studies of meta-analysis have explored the moderating effect of publication year by using meta-regression. This type of analysis could allow us to observe the hidden changing patterns of target variables, such as the significant differences of cohort (e.g., post-80s and post-00s) on the relationship between SRL and academic achievement. Thus, it is intriguing to examine if publication year would be a moderator in the meta-analysis.

## Why Chinese Students?

Although researchers have provided critical reviews of existing self-regulated learning studies (e.g., Panadero, 2017; Sahdan and Abidin, 2017), they have rarely included studies from outside Europe or North America (Richardson et al., 2012). In fact, because of language issues, few studies conducted in Asia were located and Asian effect sizes were omitted from the meta-analysis. In addition, education does not take place in a vacuum, the results of education will naturally differ with different culture in different environments. Many studies have found students in East Asia outperformed their counterparts in the West in mathematic achievement (Mullis et al., 2000, 2004; Jerrim and Shure, 2016). However, teaching methods in East Asia are not perceived as advanced as in Western countries (Leung et al., 2006). For example, China is very often content oriented and examination driven. Large class size is the norm, and classroom teaching is often conducted in a whole class setting. The parity between the high math performance and a lack of advanced teaching methods is puzzling (Leung et al., 2006). It prompts for a call for further studies about the learning process of Chinese students, such as how they use self-regulated strategies to help their study. Hence, the present study includes empirical self-regulated learning studies from China (published in Chinese journals), attempting to provide an Asian insight in this field, and to understand the relationship between self-regulated learning and academic achievement of elementary and secondary education in China.

## Research Questions and Hypotheses

Based on the reasons mentioned above, we proposed several research questions in the present study. First, what are the most and least effective self-regulated learning strategies for Chinese students? Second, according to Zimmerman's cyclical model of self-regulated learning, which SRL phase has the largest effect size on academic achievement? Third, are the moderating effects of gender, educational stage, and publication year significant? How do they impact the relationship between self-regulated learning and academic achievement? The following hypotheses were proposed.

1. The effect size of SRL on academic achievement would be significant for primary and secondary school students in China.
2. Significant differences would be found for the effect sizes between different phases and sub-processes of SRL.
3. The relationship between SRL and academic achievement in high school students may be stronger than that of elementary school students.
4. The effect sizes of SRL on academic achievement in girls are larger than those of boys.

## Methods

### Literature Search

The present meta-analysis aimed to get conclusions from real teaching situations in China, and only Chinese journals were considered to narrow down the focus. Empirical studies were searched using the CNKI (China National Knowledge Infrastructure), Wanfang Database, and Vip Paper Check System. The following key words were used: “Self-regulated learning,” “self-monitoring,” “self-management,” “time management,” “learning strategies,” “metacognition,” “goal orientation,” “goal setting,” “self-efficacy,” “self-reflection,” “self-evaluation”; “academic achievement,” “interim/final grade,” “academic performance,” “grades,” and “academic success.” Then, we also searched through the reference lists of systematic review articles of SRL.

### Inclusion and Exclusion Criteria

**Figure 1:**
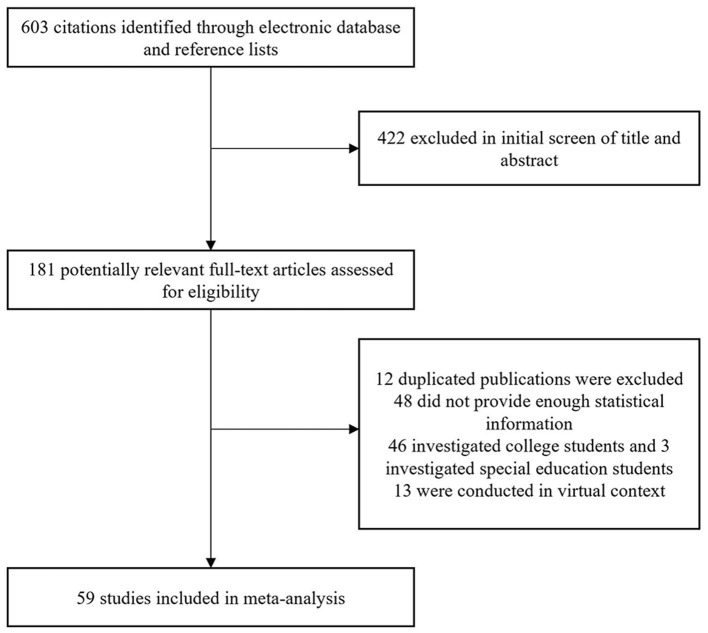
Flow diagram of papers included in the present study.

After literature searching, all the authors discussed the following inclusion and exclusion criteria: (1) In order to ensure ecological validity, empirical studies should be conducted in real teaching situations. (2) Participants in the empirical studies must be elementary, junior high, or senior high school students in China. Undergraduate students and students of special education were excluded. (3) Empirical studies based on the online learning environments were excluded. (4) Empirical studies should report the correlation coefficients, sample sizes, means, and standard deviations. After literature screening (See Figure 1), 59 empirical studies (i.e., 55 cross-sectional studies and 4 intervention studies) were collected (Detailed information of these studies can be found in the Appendix).

### Coding of Studies

We coded the studies and computed effect size using Comprehensive Meta-Analysis version 2 (Borenstein et al., 2005). The following information from each paper were coded: (1) basic information (title of the study, publication year and journal); (2) type of SRL strategies; (3) type of academic achievement, including language performance (Chinese and English), science performance (mathematics and physics), and integrated performance (i.e., when the study did not specify disciplines but use integrated interim or final grades); (4) educational stage, including elementary school, junior high school, and senior high school; (5) proportion of female; (6) phase of SRL, which was classified into forethought phase, performance phase, or self-reflection phase according to Zimmerman's (2008) cyclical model of SRL. For the cross-sectional studies (55 studies), the correlation coefficient and sample size were extracted to calculate the effect size. As for the intervention studies (only 4 studies), sample sizes, means, and standard deviations in experimental group and control group were extracted.

All articles were separately coded by the first author and the second author. Inter-rater agreement CA = 2^*^(59-14)/(59+59) = 0.763. Because the two authors had different opinions about the classification of SRL strategies, they did not reach a consensus on 14 of the 59 studies. In cases where classification was not in agreement, the two authors discussed and came to a consensus.

### Fidelity of Intervention Studies

Although most studies in the current meta-analysis were cross-sectional studies, 4 out of the 59 studies were based on intervention. It is necessary to report treatment fidelity of these intervention studies to ensure that study outcomes were due to treatment rather than factors incidental to the intervention (Díaz-Prieto and García-Sánchez, 2015; Canedo-García et al., 2017). Table 1 showed the treatment fidelity of the four intervention studies in the meta-analysis. Specifically, one study trained intervention providers. Two studies had clear intervention protocol. One study used interview to check the efficacy of intervention. Pre-post measures were used by three studies. All studies have the record of the sessions.

**Table 1:** Compliance with EBI controls by the four intervention studies.

**N**	**Training intervention providers**	**Protocol**	**Pre-post measures**	**Number of groups**	**Recording of sessions**	**Interview check**
4	1	2	3	All studies used two groups	4	1

### Effect Size and Publication Bias

Most empirical studies reported correlations, so the present meta-analysis used *r* to indicate effect size. Then we transformed *r* into Cohen's *d* (i.e., standardized mean difference). A few studies reported the means, sample sizes, and standard deviations of experimental group and control group, we also transformed these number into Cohen's *d*. In interpreting the effect size, we followed Rosenthal (1996)'s guidelines (*d* = 0 ~ 0.29, small; *d* = 0.30 ~ 0.79, medium, *d* = 0.80 ~ 1.29, large). The current meta-analysis was conducted with a random effects model, because accumulated evidence suggested heterogeneity in effect sizes (National Research Council, 1992), and we wished to draw inferences beyond the set of studies included in the analysis (Hedges and Vevea, 1998; Borenstein et al., 2010).

Journals tend to accept articles that report significant results, which may probably cause publication bias. We conducted analyses to address the possibility that the results might be affected by publication bias. Funnel plot, fail-safe *N*, and trim and fill procedures were used to examine publication bias. The horizontal axis of funnel plot stands for effect size, and the vertical axis of funnel plot stands for standard error. In the presence of publication bias, the bottom of the plot would tend to show a higher concentration of studies on one side of the mean than the other. Fail-safe *N* computes the number of missing studies that would need to be added to the analysis to yield a statistically non-significant overall effect. Rothstein et al. (2006) suggested if fail-safe *N* is less than 5*k*+10, meta-analysis would exist publication bias. Trim and fill procedures were implemented to impute studies on the funnel plot and keep the plot symmetric (Duvall and Tweedie, 2000).

## Results

### SRL Strategies and Academic Achievement

Two hundred and sixty-three independent samples reported the correlations between specific SRL strategies (e.g., attribution or goal setting) and academic achievement. Five independent samples viewed SRL as an integrated conception and reported the correlations between SRL and academic performance. Table 2 displayed the effect sizes of ten SRL strategies. The effect sizes of self-efficacy, self-evaluation, and task strategies were relatively larger than other strategies. The effect sizes of attribution and goal orientation were relatively small. Given that the sample size of goal setting, integrated (i.e., the independent samples that did not distinguish specific SRL strategy), and self-satisfaction were smaller than 8 (Borenstein et al., 2009), further discussion about these strategies were omitted. When all the empirical studies were pulled together, the effect size of SRL was 0.435. Heterogeneity test found that there was considerable heterogeneity across the independent samples [*Q*_(263)_ = 3,766.115, *p* < 0.001, *I*^2^ = 93.0%], suggesting the presence of moderators.

**Table 2:** Mean effect sizes of SRL strategies.

**SRL strategies**	***k***	**Cohen's *d***	**95% CI**	***Z***	***p***
Attention focus	8	0.537	0.189–0.885	3.024	0.002
Attribution	8	0.272	−0.003–0.547	1.942	0.052
Goal orientation	68	0.092	0.032–0.152	2.997	0.003
Goal setting	4	0.474	0.311–0.636	5.716	0.000
Integrated	5	1.018	0.284–1.752	2.717	0.007
Metacognitive monitoring	43	0.388	0.316–0.460	10.530	0.000
Self-efficacy	39	0.699	0.586–0.812	12.133	0.000
Self-evaluation	13	0.717	0.430–1.004	4.892	0.000
Self-satisfaction	3	−0.033	−0.965–0.899	−0.070	0.944
Task Interest/value	9	0.405	0.022–0.787	2.075	0.038
Task strategies	64	0.600	0.518–0.682	14.379	0.000
SRL (pooled)	264	0.365	0.392–0.401	19.945	0.000

*All the effect sizes were based on random effects analysis in the present study; Integrated stands for the independent samples that did not distinguish specific SRL strategy. In interpreting the effect size, we followed Rosenthal (1996)'s guidelines (d = 0 ~ 0.29, small; d = 0.30 ~ 0.79, medium, d = 0.80 ~ 1.29, large)*.

Table 3 shows the mean effect sizes grouped according to disciplines. Specifically, the effect size of science (including performances of mathematics and physics) was relatively higher than that of language (including performances of Chinese and English). There were many empirical studies did not specify disciplines, but used integrated interim or final grades to measure students' academic achievement. These independent samples were labeled as integrated. The mean effect size of the integrated performance was 0.592.

**Table 3:** Mean effect sizes grouped according to the group of discipline.

**Discipline**	***k***	**Cohen's *d***	**95% CI**	***Q_***B***_***	***df***	***p***
Science	59	0.449	0.377–0.522	22.412	2	0.000
Language	80	0.292	0.231–0.352			
Integrated	125	0.592	0.465–0.719			

*Integrated stands for the integration of mathematics performance and language performance*.

### Phases of SRL and Academic Performance

According to Zimmerman's theory, we classified SRL strategies into forethought phase, performance phase, and self-reflection phase. Concretely, attention focus, goal orientation, goal setting, and task interest/value were classified into the forethought phase. Task strategies, metacognitive monitoring, and attention focus were classified into the performance phase. Attribution, self-satisfaction/affect, and self-evaluation were classified into the self-reflection phase. Table 4 showed that the differences among the three phases were significant [*Q*_*B*_(3) = 22.025, *p*_*B*_ = 0.000]. The effect sizes of forethought phase, performance phase and self-reflection phase were 0.316, 0.525, and 0.465.

**Table 4:** Mean effect sizes grouped according to the phases of SRL.

**SRL phases**	***k***	**Cohen's *d***	**95% CI**	***Q_***B***_***	***df***	***p_***B***_***
Forethought	119	0.316	0.245–0.386	22.025	3	0.000
Performance	115	0.525	0.464–0.585			
Self-reflection	25	0.465	0.261–0.669			
Integrated	5	1.018	0.284–1.752			

*Integrated stands for the independent samples that did not distinguish specific SRL strategy, these samples could not be classified to a specific phase of SRL*.

### Moderator Analyses

All independent samples were classified into three groups: elementary school, junior high school, and senior high school. Some independent samples included both elementary school students and junior high school students, or junior high school students and senior high school students, but did not specify the educational stage. These independent samples were not included in this moderator analysis. It was found that educational stage significantly moderated the relationship between SRL and academic performance [*Q*_*B*_(2) = 38.623, *p*_*B*_ < 0.001, See Table 5]. Specifically, the effect size of junior high school students (Cohen's *d* = 0.629) was higher than that of senior high school students (Cohen's *d* = 0.303) and elementary school students (Cohen's *d* = 0.164).

**Table 5:** Mean effect sizes grouped according to educational stage.

**Educational stage**	***k***	**Cohen's *d***	**95% CI**	***Q_***B***_***	***df***	***p_***B***_***
Elementary school	19	0.164	0.038–0.289	38.623	2	0.000
Junior high school	84	0.629	0.527–0.732			
Senior high school	81	0.303	0.231–0.375			

*Some independent samples included both elementary school students and junior high school students, or junior high school students and senior high school students, but did not distinguish specific educational stage. These independent samples were not included in this analysis*.

**Figure 2:**
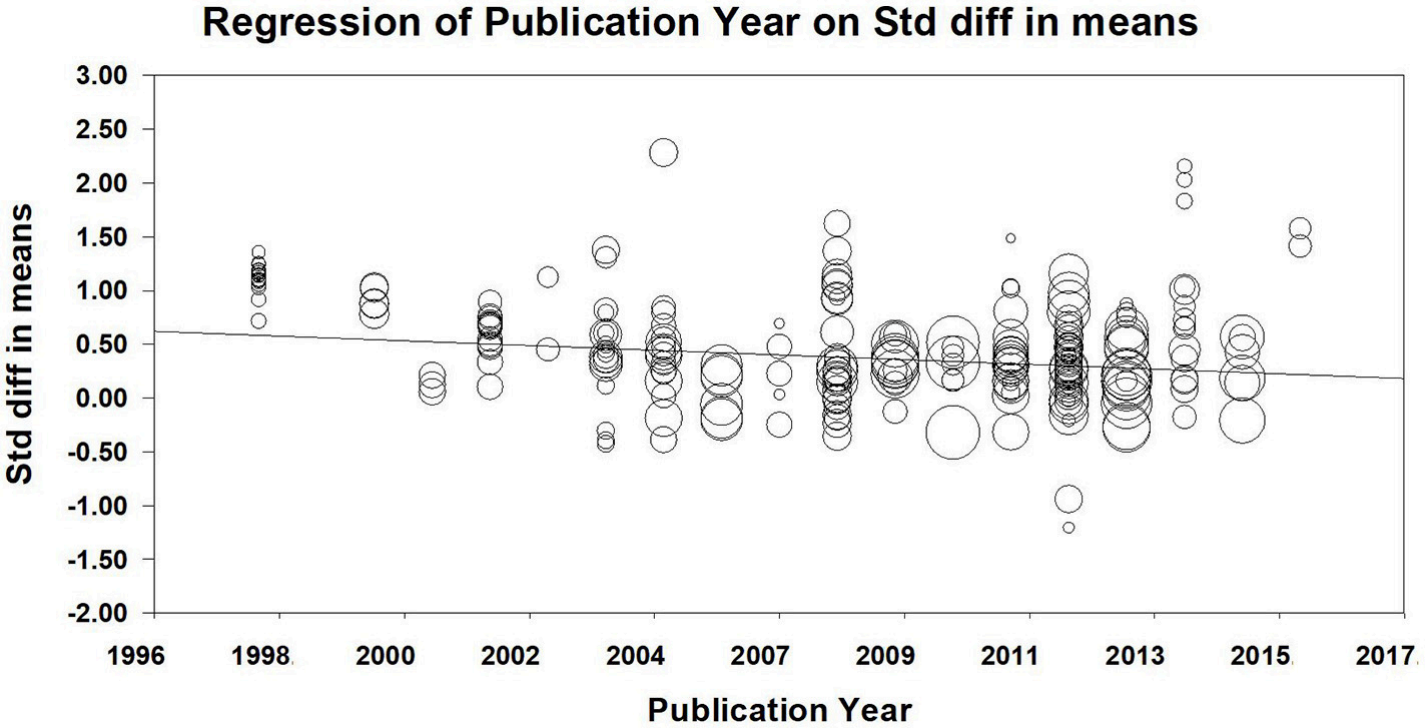
The moderating effect of publication year.

The time span of the present meta-analysis ranged from 1998 to 2016. We examined the moderating effects of publication year and the proportion of female using meta-regression. From 1998 to 2016, the effect size of SRL gradually declined [*Slope* = −0.020, *Q*_*model*_(1) = 147.470, *p* < 0.001. See Figure 2]. The moderating effect of the proportion of female was not found [*Slope*_*FisherZ*_ = 0.001, *Q*_*model*_(1) = 0.273, *p* = 0.601; *Slope*_*d*_ = 0.001, *Q*_*model*_(1) = 49.642, *p* < 0.001].

### Publication Bias

**Figure 3:**
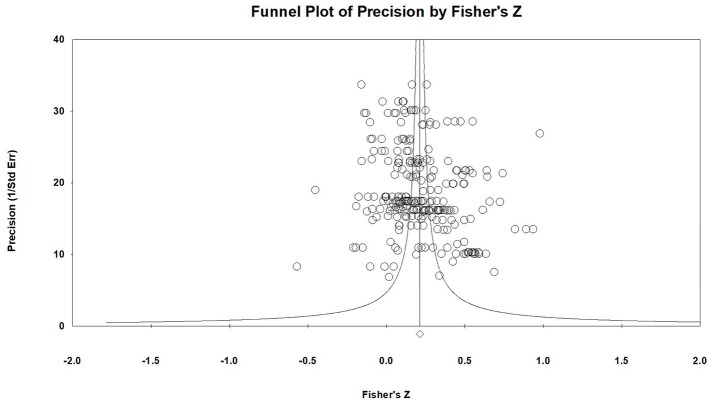
Funnel plot.

As is shown in Figure 3, there were almost no low accurate and high effect size studies in the funnel plot (bottom right), and the whole plot presents symmetry. In addition, the fail-safe *N* of the present study was 9,558. According to Rosenthal's (1996) suggestion, if the fail-safe *N* is smaller than 5*k* +10, then the meta-analysis may have publication bias. In the presence of publication bias, studies are expected to be systematically missing in a manner that can be identified by the trim and fill analyses (Duvall and Tweedie, 2000). In the present meta-analysis, the trimmed studies were 0. In summary, the results were not affected by publication bias.

## Discussion

The present study used meta-analysis to examine data from 59 empirical studies (264 independent samples), covering 23,497 Chinese students, and tried to understand the relationship between SRL strategies and academic achievement in education in China. We examined the moderating effects of gender, grade, and year of publication. According to Rosenthal (1996)'s guidelines for effect size, analyses found that medium effect sizes of self-evaluation, self-efficacy, task strategies, which were relatively larger than other strategies. In contrast, the effect size of attribution and goal orientation were small but present. Basically, Hypothesis 1 was supported by these results. From the perspective of discipline, the effect size of SRL on the academic performance of science (medium) was higher than that of language (small). Moreover, the effect size of forethought phase was smaller than the other two phases, which suggested that performance and self-reflection were critical phases of SRL. This result lent support to Hypothesis 2. The moderating effects of grade and publication year were significant. The effect size of junior high school was larger than that of senior high school, and the effect size of elementary school was small, suggesting that Hypothesis 3 was supported. The moderating effect of publication year was found, with the relationship between SRL and academic gradually weakened from 1998 to 2016.

### The Effective SRL Strategies for Chinese Students

We found that the effect sizes of self-efficacy, self-evaluation, and task strategies were relatively large, suggesting that these were important SRL strategies for Chinese students. Similar to the present study, Richardson et al. (2012) conducted a meta-analysis including 241 unique data sets from Europe and North America, and found that performance self-efficacy was strongly associated with GPA, comprising the largest effect observed. The effect size of strategic approach to learning was also larger than that of peer learning, learning goal orientation, and performance goal in their study. The meta-analysis of Dignath et al. (2008) also reported that students' self-efficacy represented the largest correlation with academic performance, and the effect sizes of goal setting, goal orientation, and attribution were relatively small. Task strategies (e.g., cognitive learning strategies) were also found to be effective for Korean learners in the previous meta-analytic research, which had a significantly large effect size (Kim et al., 2008a). Kizilcec et al. (2017) found strategic planning predicted attainment of personal course goals. These previous findings, together with results in the present meta-analysis, suggest that having knowledge and skill does not produce high-quality problem solving if learners lack the self-efficacy to use their personal resources. Task strategies help learning or problem solving by reducing a task to its essential parts and reorganizing them meaningfully. Self-evaluation compares self-monitored outcomes with learners' goal or other standards. Each element may represent the essential function of its corresponding phase, and thus exhibit a large effect size in terms of its association to the academic achievement. Of course, this is not to say that goal orientation and attribution are unnecessary strategies. In fact, many definitions and theories have emphasized the importance of goal orientation and attribution (Cook and Artino, 2016; Latham et al., 2016). But in relative terms, self-efficacy, task strategies, and self-evaluation were more effective than goal and attribution. Therefore, in real teaching situations, educators should know how to improve students' self-efficacy, and teach them a few useful task strategies (e.g., cognitive strategies). Teachers also should guide students to evaluate their performance and to understand their strengths and weaknesses.

In addition, the present study found different effect sizes for the different academic fields (higher effects for science performance than for language), suggesting that SRL strategies were more effective for improving science performance. Lucangeli and Cornold (1997) suggested that metacognition seems to be very important to gain a better understanding of successful mathematical performance. The usage of SRL strategies may vary in different disciplines (Bembenutty et al., 2013). Wolters and Pintrich (1998) explored the use of SRL strategies of junior high school students in three academic subjects (i.e., mathematics, English, and social studies), and different subjects were reported to be different in value and interest for self-efficacy, academic tasks and test anxiety. The demand of cognitive ability in mathematics is higher than those of linguistics and social science (Loong, 2012), and cognitive ability closely related to SRL. Metallidou and Vlachou (2010) examined the SRL profile of fifth and sixth grade students who were differentiated in their task value beliefs in mathematics and language, and found that teachers' ratings for their students' knowledge and use of strategies significantly predicted students' mathematics performance, but not language performance. The results indicated a domain-specific aspect of SRL strategies.

### The Critical Phases of SRL for Chinese Students

According to the phases and sub-processes of SRL, we classified all SRL strategies in empirical studies into forethought phase, performance phase, and self-reflection phase. The effect size of forethought phase (small) was lower than that of performance phase (medium) and self-reflection phase (medium), but there was no difference between performance phase and self-reflection phase.

From a theoretical perspective, researchers have different views in terms of whether SRL should contain the forethought phase (including motivation, goal setting, planning, and the like). For example, social cognitive view suggested that SRL is acquired through a triadic interaction between three characteristics: (1) self-observation (monitoring one's actions); (2) self-judgement (evaluation of one's performance); (3) self-reactions (one's response to performance outcomes), which did not involve the elements of motivation or planning (Broadbent and Poon, 2015). Miller and Brown (1991) proposed that SRL have seven procedures, covering information input, self-evaluation, instigation to change triggered by perceptions of discrepancy, search for ways to reduce discrepancy, planning for change, implementation of behavior change, and evaluation of progress toward a goal. Their model began to pay attention to goal setting and planning, but not motivation. Zimmerman's phases and sub-processes of SRL incorporated the elements of motivation, goal setting, and planning, all the three elements constituted the forethought phase. Thus, the disagreement between these theories focused on the forethought phase. Nevertheless, the aforementioned researchers all agreed that the cores of SRL are the process of self-monitoring and the execution of self-control behavior. The present meta-analysis found that the effect size of the forethought phase was lower than performance phase and self-reflection phase, suggesting that the core phases of SRL have a closer association with academic achievement. Based on the SRL theories and the results of the present study, the critical phases of SRL for Chinese students should be the performance phase and the self-reflection phase.

### The Moderating Effects of Education Stage and Publication Year

Results of the present study indicates that the moderating effect of grade was significant, whereas the effect size of elementary school students was smaller than senior high school and junior high school. It could be because the development of motivation and self-efficacy of elementary school students is insufficient, and their SRL motivation, emotion and self-efficacy still stay fuzzy (Su et al., 2013). The effect size of junior high school students was two times larger than that of senior high school students. The cognitive development of junior high school students has already been in formal operational stage, and their abstract thinking ability is close to adult. Junior high school is also an important development stage of SRL (Nurjanah and Dahlan, 2018). However, we found that the relationship between SRL and academic achievement was decreasing from junior high school to senior high school. This might be because the speed of SRL development declines with age (Ji, 2005). The curriculum of senior high school is more difficult than junior high school, and the development of SRL also slows down. Moreover, there are many other factors that influence students' academic performance, such as teaching quality, curriculum difficulty, reasoning, problem solving, and peer relationships (Hernández et al., 2017; Llorca et al., 2017; Stadler et al., 2017). These factors may have enhancing effects on academic achievement, while the impact of SRL declines in high school.

From 1998 to 2016, the effect size of SRL gradually decreased, suggesting that the moderating effect of publication year was significant. One possible interpretation is the birth cohort effect (Xin and Chi, 2008). The earliest empirical study in the present meta-analysis was published in 1998, and the latest study was published in 2016. Middle school students in 1998 (post-80s) and in 2016 (post-00s) experienced different social and cultural environments. The most significant social change in China from 1998 to 2016 is probably the popularity of the Internet (CNNIC, 1996, 2016). The rapid and global expansion of the Internet has transformed the way our world operates and the way people think and interact (Gosling and Mason, 2015). Previous studies found that self-control (closely related to SRL) was associated with problematic Internet use among Chinese adolescents (He et al., 2012; Sun et al., 2015; Mei et al., 2016). Lack of self-control is one of the major characteristics of problematic use of Internet (Kim et al., 2008b). Internet is more accessible to post-00s (who are usually digital natives) than to post-80s (who are digital immigrants) during their high school study. Ransdell (2010) found that older cohorts of learners can be better online learners. Digital natives (i.e., Millennial students) showed poorer knowledge application skill and were more self-reliant than older students (Ransdell et al., 2011). Digital natives may use a resource of the online learning environment only if they deem it necessary; or else they may need extra encouragement to participate actively in a course and to exchange information with their peers (Ransdell et al., 2011). These evidences may help to explain the moderating effect of publication year in our study. However, the negative effect of Internet on self-control is only one of possible interpretations. It should be cautious when interpret the moderating effect of publication year on the association of SRL and academic achievement.

### Limitations and Directions for Future Research

Some limitations to the current meta-analysis should be noted. First, the sample sizes of some SRL strategies were small, such as of self-satisfaction and goal setting, so we could not provide further discussion on these strategies. Moreover, few empirical studies reported social economic status (SES), which might be an important potential moderator (see, e.g., Pintrich, 2004) but cannot be analyzed in the present study.

Second, few SRL studies in China were conducted in online learning environments, hence the difference between traditional learning and online learning could not be analyzed. We also exclude the studies from special education. Compared to traditional classroom students, SRL ability to control, manage, and plan learning actions is particularly important to online learners (Ally, 2004). The online learning conditions were less likely to be instructor-directed than they were to be learner-directed. Independent learning and collaborative learning are significant features of online learning (Adam et al., 2017). It is surmised that SRL is more important for online learning than traditional learning, which may yield different effect size.

Third, we did not evaluate the effects of the specific methodological controls of the included studies (i.e., studies conducted in real teaching situation) on the relationship between SRL and academic achievement. For example, the research design (e.g., experimental or quasi-experimental design) and fidelity of data collection may be different in the included studies. In addition, most empirical studies in the current meta-analysis were cross-sectional studies, whereas only four studies were based on intervention, which limited the generalizability of the current study to some extents. We evaluated the treatment fidelity of these intervention studies but found that few studies provided a rigorous treatment fidelity control. Despite these limitations, the current study provided an insight from Chinese scientific community to the field of SRL.

Future studies could examine the moderating effect of SES or other interesting variables. More SRL empirical studies in online learning are needed in China, so researchers could compare the difference effects between traditional learning and online learning on the relationship between SRL and academic performance. For example, Broadbent and Poon (2015) suggested that online learners should not dedicate time to using elaboration, rehearsal, and organizing when learning new curriculum as these SRL strategies may not increase the possibility of academic success. Specifically, massive open online courses (MOOCs) have attracted much attention from educators and students in the recent years, such courses may need high level SRL abilities to achieve academic success. Additionally, education technologies, such as educational games and intelligent tutoring system, develop fast in the last decade. From 2004 to 2016, the new media consortium predicted educational games, intelligent tutoring system, and virtual assistant could be important new technologies that impact higher education deeply. Future studies could analyze SRL strategies in educational games or explore whether immersion and other characteristics in games could promote students' SRL ability.

## Conclusions and Implications

The conclusions emerging from the present study are as follows: First, for Chinese elementary school students and high school students, self-efficacy, task strategies, and self-reflection turned to be better SRL strategies. Second, SRL strategies were more effective for science disciplines than for linguistics disciplines. Third, the performance phase and the self-reflection phase are critical phases of SRL in that they have a larger effect on academic achievement. Fourth, the effect size of junior high school was larger than that of senior high school and elementary school. Junior high school is probably the critical period of SRL development. Fifth, from 1998 to 2016, the effect size of SRL was gradually decreasing. Lastly, most empirical studies in the present meta-analysis were cross-sectional studies, whereas only four were intervention studies. More empirical-based intervention studies may be necessary before generalizing the current conclusions to educational practice.

According to the present study, educators should improve students' self-efficacy, and teach them some sample task strategies. Teachers could encourage students to evaluate their own performance and to reflect their learning actions. It could be beneficial for teachers to know how to teach SRL at different educational levels and disciplines, because SRL works better at different educational levels and disciplines. At the same time, teachers need to receive training on SRL theory and models to understand how they can maximize their students' learning (Panadero, 2017). For example, new teachers need to receive pedagogical training for their future adaptation to the workplace. In-service teachers also need to receive training on SRL as they most probably did not receive any during their pre-service preparation (Moos and Ringdal, 2012). In addition, this study sheds light on how Chinese students use SRL in their learning. Self-efficacy, task strategies, and self-evaluation were the most important SRL strategies for Chinese students, whereas self-efficacy, grade goal setting, and effort regulation are particularly important for learners in Western countries (Richardson et al., 2012). It seems that students in China show a different SRL pattern, which may help to preliminarily explain the parity between the high performance (i.e., math) and a lack of advanced teaching methods in China (Leung et al., 2006). However, there is still need for experimental and longitudinal studies exploring the answers for this question in the future.

## Author Contributions

JL searched for references, coded and analyzed the data, and prepared the manuscript. HY coded the data and helped prepare the manuscript. YT designed the study and prepared the manuscript. ZZ and XH supervised the whole research process and proofread the manuscript.

### Conflict of Interest Statement

The authors declare that the research was conducted in the absence of any commercial or financial relationships that could be construed as a potential conflict of interest.
