# Supplementary material for: What Are the Effects of Self-Regulation Phases and Strategies for Chinese Students? A Meta-Analysis of Two Decades Research of the Association Between Self-Regulation and Academic Performance
Source: Front Psychol. 2018 Dec 18;9:2434. doi: 10.3389/fpsyg.2018.02434 (PMC6305361; doi:10.3389/fpsyg.2018.02434)
Supplement: Supplementary file 1 [file Data_Sheet_1.pdf]

Appendix

Table A.1 Summary of coding information of the 55 cross-sectional studies and 4 intervention studies.

Independent sample ID	Publication year	SRL Phases	SRL strategies	Discipline	Educational stage	Correlation	Sample size	Percentage of female
**Cross-sectional Studies**								
An, M., & Yu, J.Y_1	2009	Performance	Metacognitive_monitoring	Integrated	Junior_Senior	0.158	910	35.78
An, M., & Yu, J.Y_2	2009	Performance	Metacognitive_monitoring	Integrated	Senior	0.101	482	NA
An, M., & Yu, J.Y_3	2009	Performance	Metacognitive_monitoring	Integrated	Junior	0.271	428	NA
An, M., & Yu, J.Y_4	2009	Performance	Metacognitive_monitoring	Language	Junior_Senior	0.172	910	35.78
An, M., & Yu, J.Y_5	2009	Performance	Metacognitive_monitoring	Science	Junior_Senior	0.154	910	35.78
An, M., & Yu, J.Y_6	2009	Performance	Metacognitive_monitoring	Language	Junior_Senior	0.111	910	35.78
Cao, L. R., & Cao, X. H_1	2004	Forethought	Self-efficacy	Language	Senior	0.201	123	46.34
Cao, L. R., & Cao, X. H_2	2004	Performance	Metacognitive_monitoring	Language	Senior	0.057	123	46.34
Cao, L. R., & Cao, X. H_3	2004	Forethought	Task_interest/value	Language	Senior	-0.208	123	46.34
Cao, L. R., & Cao, X. H_4	2004	Forethought	Self-efficacy	Language	Senior	0.289	123	46.34
Cao, L. R., & Cao, X. H_5	2004	Performance	Metacognitive_monitoring	Language	Senior	0.241	123	46.34
Cao, L. R., & Cao, X. H_6	2004	Forethought	Task_interest/value	Language	Senior	-0.193	123	46.34
Cao, L. R., & Cao, X. H_7	2004	Forethought	Self-efficacy	Science	Senior	0.37	123	46.34
Cao, L. R., & Cao, X. H_8	2004	Performance	Metacognitive_monitoring	Science	Senior	0.221	123	46.34
Cao, L. R., & Cao, X. H_9	2004	Forethought	Task_interest/value	Science	Senior	-0.15	123	46.34
Chang, H. Q., & Pan, Y_1	2013	Forethought	Self-efficacy	Integrated	Junior_Senior	0.08	183	53.01
Chang, H. Q., & Pan, Y_2	2013	Performance	Metacognitive_monitoring	Integrated	Junior_Senior	0.37	183	53.01
Chang, H. Q., & Pan, Y_3	2013	Forethought	Task_interest/value	Integrated	Junior_Senior	0.35	183	53.01
Chen, C., Gong, S. Y., & Chai, X. Y_1	2013	Forethought	Goal_orientation	Science	Junior_Senior	-0.14	888	43.81
Chen, C., Gong, S. Y., & Chai, X. Y_2	2013	Forethought	Goal_orientation	Science	Junior_Senior	0.06	888	43.81
Chen, C., Gong, S. Y., & Chai, X. Y_3	2013	Forethought	Goal_orientation	Science	Junior_Senior	-0.13	888	43.81
Chen, C., Gong, S. Y., & Chai, X. Y_4	2013	Forethought	Goal_orientation	Science	Junior_Senior	0.05	888	43.81
Chen, C., Gong, S. Y., & Chai, X. Y_5	2013	Forethought	Goal_orientation	Science	Junior_Senior	0.01	888	43.81
Chen, C., Gong, S. Y., & Chai, X. Y_6	2013	Forethought	Goal_orientation	Science	Junior_Senior	0.12	888	43.81

Independent sample ID	Publication year	SRL Phases	SRL strategies	Discipline	Educational stage	Correlation	Sample size	Percentage of female
Chen, J. J., & Li, S. F_1	2012	Reflection	Self-satisfaction/affect	Integrated	Junior	-0.319	364	45.329
Chen, J. J., & Li, S. F_2	2012	Reflection	Self-satisfaction/affect	Integrated	Junior	0.425	364	45.329
Chen, J. J., & Li, S. F_3	2012	Reflection	Attribution	Integrated	Junior	0.273	364	45.329
Chen, J_1	2009	Forethought	Goal_orientation	Integrated	Senior	0.068	235	46
Chen, J_2	2009	Forethought	Goal_orientation	Integrated	Senior	-0.063	235	46
Chen, J_3	2009	Forethought	Goal_orientation	Integrated	Senior	0.283	235	46
Chen, J_4	2009	Forethought	Goal_orientation	Integrated	Senior	0.127	235	46
Chen, L. J_1	2011	Pooled	Integrated	Integrated	Senior	0.273	534	NA
Chen, L. J_2	2011	Forethought	Self-efficacy	Integrated	Senior	0.374	534	NA
Chen, L. J_3	2011	Forethought	Self-efficacy	Integrated	Senior	0.149	534	NA
Chen, L. J_4	2011	Forethought	Goal_orientation	Integrated	Senior	0.012	534	NA
Chen, L. J_5	2011	Forethought	Goal_orientation	Integrated	Senior	-0.156	534	NA
Chen, L. J_6	2011	Forethought	Goal_orientation	Integrated	Senior	0.15	534	NA
Chen, M. G., & Hu, Z. B_1	2008	Performance	Task_strategies	Integrated	Senior	0.19	270	51.48
Chen, M. G., & Hu, Z. B_2	2008	Performance	Task_strategies	Integrated	Senior	0.17	270	51.48
Chen, M. G., & Hu, Z. B_3	2008	Performance	Task_strategies	Integrated	Senior	0.08	270	51.48
Chen, M. G., & Hu, Z. B_4	2008	Forethought	Goal_orientation	Integrated	Senior	-0.09	270	51.48
Chen, M. G., & Hu, Z. B_5	2008	Forethought	Goal_orientation	Integrated	Senior	-0.04	270	51.48
Chen, M. G., & Hu, Z. B_6	2008	Forethought	Goal_orientation	Integrated	Senior	0.09	270	51.48
Chi, Y. K., & Zhou, G. T_1	2003	Forethought	Self-efficacy	Integrated	Junior	0.49	227	50.66
Chi, Y. K., & Zhou, G. T_2	2003	Performance	Task_strategies	Integrated	Junior	0.22	227	50.66
Chi, Y. K., & Zhou, G. T_3	2003	Performance	Task_strategies	Integrated	Junior	0.22	227	51
Cui, N., Liu, Y. Y., & Gao, B. Y_1	2008	Forethought	Goal_orientation	Language	Senior	0.076	329	39.81
Cui, N., Liu, Y. Y., & Gao, B. Y_2	2008	Forethought	Goal_orientation	Language	Senior	-0.079	329	39.81
Cui, N., Liu, Y. Y., & Gao, B. Y_3	2008	Forethought	Goal_orientation	Language	Senior	-0.001	329	39.81
Cui, N., Liu, Y. Y., & Gao, B. Y_4	2008	Forethought	Goal_orientation	Science	Senior	0.126	329	39.81
Cui, N., Liu, Y. Y., & Gao, B. Y_5	2008	Forethought	Goal_orientation	Science	Senior	-0.175	329	39.81
Cui, N., Liu, Y. Y., & Gao, B. Y_6	2008	Forethought	Goal_orientation	Science	Senior	-0.003	329	39.81

Independent sample ID	Publication year	SRL Phases	SRL strategies	Discipline	Educational stage	Correlation	Sample size	Percentage of female
Cui, N., Liu, Y. Y., & Gao, B. Y_7	2008	Forethought	Goal_orientation	Language	Senior	0.075	329	39.81
Cui, N., Liu, Y. Y., & Gao, B. Y_8	2008	Forethought	Goal_orientation	Language	Senior	-0.008	329	39.81
Cui, N., Liu, Y. Y., & Gao, B. Y_8	2008	Forethought	Goal_orientation	Integrated	Senior	0.123	329	39.81
Cui, N., Liu, Y. Y., & Gao, B. Y_9	2008	Forethought	Goal_orientation	Integrated	Senior	-0.117	329	39.81
Cui, N., Liu, Y. Y., & Gao, B. Y_10	2008	Forethought	Goal_orientation	Integrated	Senior	0.037	329	39.81
Cui, N., Liu, Y. Y., & Gao, B. Y_11	2008	Forethought	Goal_orientation	Language	Senior	0.097	329	39.81
Ge, M, G., & Jin, Y_1	2005	Performance	Task_strategies	Language	Senior	0.2	239	44.77
Ge, M, G., & Jin, Y_2	2005	Performance	Task_strategies	Language	Senior	0.01	239	44.77
Ge, M, G., & Jin, Y_3	2005	Performance	Task_strategies	Language	Senior	0.12	239	44.77
Ge, M, G., & Jin, Y_4	2005	Performance	Task_strategies	Language	Senior	0.16	239	44.77
Ge, M, G., & Jin, Y_5	2005	Performance	Task_strategies	Language	Senior	0.23	239	44.77
Gu, S. H., Xin, T., & Li, H_1	1998	Performance	Task_strategies	Science	Junior	0.476	109	44.95
Gu, S. H., Xin, T., & Li, H_2	1998	Performance	Task_strategies	Language	Junior	0.513	105	50.48
Gu, S. H., Xin, T., & Li, H_3	1998	Performance	Task_strategies	Science	Junior	0.483	109	44.95
Gu, S. H., Xin, T., & Li, H_4	1998	Performance	Task_strategies	Science	Junior	0.511	109	44.95
Gu, S. H., Xin, T., & Li, H_5	1998	Performance	Task_strategies	Science	Junior	0.497	109	44.95
Gu, S. H., Xin, T., & Li, H_6	1998	Performance	Task_strategies	Language	Junior	0.495	109	44.95
Gu, S. H., Xin, T., & Li, H_7	1998	Performance	Task_strategies	Language	Junior	0.503	109	44.95
Gu, S. H., Xin, T., & Li, H_8	1998	Performance	Task_strategies	Language	Junior	0.528	109	44.95
Gu, S. H., Xin, T., & Li, H_9	1998	Performance	Task_strategies	Science	Junior	0.416	105	50.48
Gu, S. H., Xin, T., & Li, H_10	1998	Performance	Task_strategies	Science	Junior	0.505	105	50.48
Gu, S. H., Xin, T., & Li, H_12	1998	Performance	Task_strategies	Science	Junior	0.466	105	50.48
Gu, S. H., Xin, T., & Li, H_13	1998	Performance	Task_strategies	Science	Junior	0.457	105	50.48
Gu, S. H., Xin, T., & Li, H_14	1998	Performance	Task_strategies	Language	Junior	0.337	105	50.48
Gu, S. H., Xin, T., & Li, H_15	1998	Performance	Task_strategies	Language	Junior	0.531	105	50.48
Gu, S. H., Xin, T., & Li, H_16	1998	Performance	Task_strategies	Language	Junior	0.561	105	50.48
Gu, S. H., Xin, T., & Li, H_17	1998	Performance	Task_strategies	Language	Junior	0.48	109	44.95
Guo, W_1	2008	Forethought	Self-efficacy	Integrated	Junior_Senior	0.462	475	55.79

Independent sample ID	Publication year	SRL Phases	SRL strategies	Discipline	Educational stage	Correlation	Sample size	Percentage of female
Guo, W_2	2008	Forethought	Self-efficacy	Integrated	Junior_Senior	0.467	475	55.79
Guo, W_3	2008	Performance	Self-efficacy	Integrated	Junior_Senior	0.485	475	55.79
Guo, W_4	2008	Performance	Metacognitive_monitoring	Integrated	Junior_Senior	0.422	475	55.79
Guo, W_5	2008	Forethought	Task_interest/value	Integrated	Junior_Senior	0.293	475	55.79
Guo, W_6	2008	Performance	Attention_focusing	Integrated	Junior_Senior	0.467	475	55.79
Guo, W_7	2008	Performance	Attention_focusing	Integrated	Junior_Senior	0.418	475	55.79
Guo, W_8	2008	Performance	Attention_focusing	Integrated	Junior_Senior	0.564	475	55.79
Han, X. H., Zheng, L. N., & Liu, R. J_1	2015	Performance	Metacognitive_monitoring	Integrated	Elementary	-0.104	813	NA
Han, X. H., Zheng, L. N., & Liu, R. J_2	2015	Performance	Task_strategies	Integrated	Elementary	0.092	813	NA
Han, X. H., Zheng, L. N., & Liu, R. J_3	2015	Forethought	Self-efficacy	Integrated	Elementary	0.273	813	NA
Ji, H. Y	2005	Pooled	Integrated	Integrated	Junior_Senior	0.752	726	50
Jiang, J. C., & Liu, H. S_1	2005	Forethought	Goal_orientation	Integrated	Junior_Senior	-0.19	283	47.7
Jiang, J. C., & Liu, H. S_2	2005	Forethought	Goal_orientation	Integrated	Junior_Senior	0.13	283	47.7
Jiang, J. C., & Liu, H. S_3	2005	Forethought	Goal_orientation	Integrated	Junior_Senior	0.13	283	47.7
Jiang, M. R., Song, H. S., & Zhang, L_1	2014	Reflection	Self-evaluation	Science	Senior	0.315	186	NA
Jiang, M. R., Song, H. S., & Zhang, L_2	2014	Reflection	Self-evaluation	Science	Senior	0.675	186	NA
Jiang, M. R., Song, H. S., & Zhang, L_3	2014	Reflection	Self-evaluation	Science	Senior	0.712	186	NA
Jiang, M. R., Song, H. S., & Zhang, L_4	2014	Reflection	Self-evaluation	Science	Senior	0.733	186	NA
Jiang, Y. Z., & Zhang, H. Z	2012	Forethought	Self-efficacy	Integrated	Junior	0.23	357	46.78
Jiao, C. Z_1	2008	Forethought	Self-efficacy	Science	Junior	0.63	458	51.97
Jiao, C. Z_2	2008	Forethought	Task_interest/value	Science	Junior	0.5	458	51.97
Jin, G. Y., Gao, J. J., & Chen, Y, W_1	2012	Forethought	Self-efficacy	Science	Junior_Senior	0.5	819	47.13
Jin, G. Y., Gao, J. J., & Chen, Y, W_2	2012	Performance	Task_strategies	Science	Junior_Senior	0.37	819	47.13
Jin, G. Y., Gao, J. J., & Chen, Y, W_3	2012	Performance	Task_strategies	Science	Junior_Senior	0.44	819	47.13
Jin, G. Y., Gao, J. J., & Chen, Y,	2012	Performance	Task_strategies	Science	Junior_Senior	0.41	819	47.13

Independent sample ID	Publication year	SRL Phases	SRL strategies	Discipline	Educational stage	Correlation	Sample size	Percentage of female
W_4								
Lei, L., Wang, L., & Culjak, T_1	2001	Forethought	Goal_orientation	Integrated	Elementary	0.105	279	45.88
Lei, L., Wang, L., & Culjak, T_2	2001	Forethought	Goal_orientation	Integrated	Elementary	0.029	279	45.88
Lei, L., Wang, L., & Culjak, T_3	2001	Forethought	Goal_orientation	Integrated	Elementary	0.061	279	45.88
Li, B., & Yang, J. L_1	2004	Forethought	Self-efficacy	Integrated	Junior	0.567	438	49.32
Li, B., & Yang, J. L_2	2004	Performance	Task_strategies	Integrated	Junior	0.282	438	49.32
Li, B., & Yang, J. L_3	2004	Performance	Task_strategies	Integrated	Junior	0.167	438	49.32
Li, B., & Yang, J. L_4	2004	Performance	Task_strategies	Integrated	Junior	0.188	438	49.32
Li, B., & Yang, J. L_5	2004	Reflection	Attribution	Integrated	Junior	0.152	438	47
Li, H. X., Lin, X., & Si, J. W_1	2015	Performance	Task_strategies	Integrated	Elementary	0.07	492	47
Li, H. X., Lin, X., & Si, J. W_2	2015	Performance	Task_strategies	Integrated	Elementary	0.21	492	47
Li, J. D., An, M., He, T. T., & Ge, G. Y_1	2009	Forethought	Self-efficacy	Integrated	Junior_Senior	0.244	910	NA
Li, J. D., An, M., He, T. T., & Ge, G. Y_2	2009	Performance	Metacognitive_monitoring	Integrated	Junior_Senior	0.185	910	NA
Li, J., Zhang, J., & Zhu, L. Q_1	2011	Reflection	Self-evaluation	Language	Junior_Senior	0.185	450	46
Li, J., Zhang, J., & Zhu, L. Q_2	2011	Reflection	Self-evaluation	Science	Junior_Senior	0.053	450	46
Li, J., Zhang, J., & Zhu, L. Q_3	2011	Reflection	Self-evaluation	Language	Junior_Senior	0.13	450	46
Li, S., & Yu, X. X_1	2002	Performance	Metacognitive_monitoring	Integrated	Junior	0.317	265	50.57
Li, S., & Yu, X. X_2	2002	Performance	Metacognitive_monitoring	Integrated	Junior	0.23	265	50.57
Li, S., & Yu, X. X_3	2002	Performance	Metacognitive_monitoring	Integrated	Junior	0.333	265	50.57
Li, S., & Yu, X. X_4	2002	Performance	Metacognitive_monitoring	Integrated	Junior	0.312	265	50.57
Li, S., & Yu, X. X_5	2002	Performance	Metacognitive_monitoring	Integrated	Junior	0.164	265	50.57
Li, S., & Yu, X. X_6	2002	Performance	Metacognitive_monitoring	Integrated	Junior	0.322	265	50.57
Li, S., & Yu, X. X_7	2002	Performance	Metacognitive_monitoring	Integrated	Junior	0.408	265	50.57
Li, S., & Yu, X. X_8	2002	Performance	Metacognitive_monitoring	Integrated	Junior	0.238	265	50.57
Li, S., & Yu, X. X_9	2002	Performance	Task_strategies	Integrated	Junior	0.349	265	50.57
Li, S., & Yu, X. X_10	2002	Performance	Task_strategies	Integrated	Junior	0.245	265	50.57
Li, S., & Yu, X. X_11	2002	Performance	Task_strategies	Integrated	Junior	0.326	265	50.57
Li, S., & Yu, X. X_12	2002	Performance	Task_strategies	Integrated	Junior	0.264	265	50.57
Li, S., & Yu, X. X_13	2002	Performance	Task_strategies	Integrated	Junior	0.357	265	50.57
Li, S., & Yu, X. X_14	2002	Performance	Metacognitive_monitoring	Integrated	Junior	0.052	265	50.57

Independent sample ID	Publication year	SRL Phases	SRL strategies	Discipline	Educational stage	Correlation	Sample size	Percentage of female
Li, X. Y., & Li, J. W_1	2004	Pooled	Integrated	Science	Junior	0.379	266	40
Li, X. Y., & Li, J. W_2	2004	Performance	Task_strategies	Science	Junior	0.174	266	40
Li, X. Y., & Li, J. W_3	2004	Forethought	Goal_orientation	Science	Junior	0.289	266	40
Li, X. Y., & Li, J. W_4	2004	Forethought	Self-efficacy	Science	Junior	0.547	266	40
Li, X. Y., & Li, J. W_5	2004	Reflection	Attribution	Science	Junior	0.192	266	40
Li, Z. X., & Hu, Y. R_1	2014	Forethought	Self-efficacy	Integrated	Junior	0.459	222	54.95
Li, Z. X., & Hu, Y. R_2	2014	Forethought	Goal_orientation	Integrated	Junior	0.309	222	54.95
Li, Z. X., & Hu, Y. R_3	2014	Forethought	Goal_orientation	Integrated	Junior	-0.088	222	54.95
Li, Z. X., & Hu, Y. R_4	2014	Forethought	Goal_orientation	Integrated	Junior	0.343	222	54.95
Li, Z. X., & Hu, Y. R_5	2014	Forethought	Goal_orientation	Integrated	Junior	0.393	222	54.95
Liu, J. L., & Liu, S. X_1	2016	Forethought	Task_interest/value	Integrated	Junior	0.577	305	53.29
Liu, J. L., & Liu, S. X_2	2016	Forethought	Self-efficacy	Integrated	Junior	0.619	304	53.29
Liu, J. X., Xin, T., Huang, G. Q., & Shen, J. L_1	2000	Performance	Task_strategies	Integrated	Junior_Senior	0.364	398	51.01
Liu, J. X., Xin, T., Huang, G. Q., & Shen, J. L_2	2000	Performance	Task_strategies	Integrated	Junior_Senior	0.459	398	51.01
Liu, J. X., Xin, T., Huang, G. Q., & Shen, J. L_3	2000	Performance	Task_strategies	Integrated	Junior_Senior	0.403	398	51.01
Liu, J. X., Xin, T., Huang, G. Q., & Shen, J. L_4	2000	Performance	Task_strategies	Integrated	Junior_Senior	0.4	398	51.01
Liu, J. X., Xin, T., Huang, G. Q., & Shen, J. L_5	2000	Performance	Task_strategies	Integrated	Junior_Senior	0.455	398	51.01
Liu, T	2015	Forethought	Self-efficacy	Integrated	Junior	0.27	302	51
Ran, R., Huang, Y. Q., & Yu, J_1	2012	Forethought	Self-efficacy	Integrated	Junior	0.268	262	61.83
Ran, R., Huang, Y. Q., & Yu, J_2	2012	Forethought	Self-efficacy	Integrated	Junior	0.24	262	61.83
Ran, R., Huang, Y. Q., & Yu, J_3	2012	Forethought	Self-efficacy	Integrated	Junior	0.022	262	61.83
Wang, J. X., & Zhang, K	2008	Reflection	Attribution	Integrated	Junior	0.424	134	57.46
Wang, L. J_1	2013	Forethought	Goal_orientation	Integrated	Junior_Senior	-0.025	986	53.04
Wang, L. J_2	2013	Forethought	Goal_orientation	Integrated	Junior_Senior	0.108	986	53.04
Wang, L. J_3	2013	Forethought	Goal_orientation	Integrated	Junior_Senior	0.075	986	53.04
Wang, L. J_4	2013	Forethought	Goal_orientation	Integrated	Junior_Senior	0.104	986	53.04
Wang, L. J_5	2013	Forethought	Goal_orientation	Integrated	Junior_Senior	0.105	986	53.04
Wang, Y. X., Cao, Y. T., & Tang, X. L_1	2013	Forethought	Self-efficacy	Integrated	Senior	0.305	794	56.05

Independent sample ID	Publication year	SRL Phases	SRL strategies	Discipline	Educational stage	Correlation	Sample size	Percentage of female
Wang, Y. X., Cao, Y. T., & Tang, X. L_2	2013	Forethought	Self-efficacy	Language	Senior	0.269	794	56.05
Wang, Y. X., Cao, Y. T., & Tang, X. L_3	2013	Forethought	Self-efficacy	Science	Senior	0.232	794	56.05
Wang, Y. X., Cao, Y. T., & Tang, X. L_4	2013	Forethought	Self-efficacy	Language	Senior	0.224	794	56.05
Wang, Y. Z., Wang, S. Z., & Ou, Y. L_1	2005	Forethought	Self-efficacy	Language	Junior	0.319	263	42.59
Wang, Y. Z., Wang, S. Z., & Ou, Y. L_2	2005	Forethought	Self-efficacy	Science	Junior	0.369	263	42.59
Wang, Y. Z., Wang, S. Z., & Ou, Y. L_3	2005	Forethought	Self-efficacy	Language	Junior	0.263	263	42.59
Wang, Y. Z., Wang, S. Z., & Ou, Y. L_4	2005	Forethought	Self-efficacy	Integrated	Junior	0.387	263	42.59
Wei, X. C., & Zhang, Y. F_1	2014	Forethought	Self-efficacy	Language	Senior	0.18	296	63.18
Wei, X. C., & Zhang, Y. F_2	2014	Reflection	Attribution	Language	Senior	0.088	296	63.18
Wu, J. Y, Hu, X. L, & Peng, J. S_1	2011	Forethought	Self-efficacy	Science	Senior	0.026	141	46.1
Wu, J. Y, Hu, X. L, & Peng, J. S_2	2011	Forethought	Self-efficacy	Science	Senior	0.459	141	46.1
Xu, L. L. & Guo, W_1	2007	Forethought	Goal_orientation	Language	Senior	-0.123	259	44.01
Xu, L. L. & Guo, W_2	2007	Forethought	Goal_orientation	Language	Senior	0.113	259	44.01
Xu, L. L. & Guo, W_3	2007	Forethought	Goal_orientation	Language	Senior	0.233	259	44.01
Yao, C. L	2013	Performance	Task_strategies	Language	Senior	0.262	611	63.99
Yao, D. W. Yan, L. F. Musa, A. & Liu, G	2011	Performance	Task_strategies	Integrated	Junior	0.219	416	57.7
Yuan, L. X_1	2005	Performance	Task_strategies	Integrated	Junior_Senior	0.253	545	55.96
Yuan, L. X_2	2005	Forethought	Self-efficacy	Integrated	Junior_Senior	0.209	545	55.96
Yuan, L. X_3	2005	Forethought	Goal_orientation	Integrated	Junior_Senior	-0.093	545	55.96
Yuan, L. X_4	2005	Forethought	Goal_orientation	Integrated	Junior_Senior	0.079	545	55.96
Yuan, L. X_5	2005	Forethought	Goal_orientation	Integrated	Junior_Senior	0.194	545	55.96
Zhang, F & Liu, C_1	2012	Performance	Metacognitive_monitoring	Integrated	Junior_Senior	0.22	308	57.47
Zhang, F & Liu, C_2	2012	Performance	Metacognitive_monitoring	Language	Junior_Senior	0.19	308	57.47
Zhang, F & Liu, C_3	2012	Performance	Metacognitive_monitoring	Science	Junior_Senior	0.23	308	57.47
Zhang, F & Liu, C_4	2012	Performance	Metacognitive_monitoring	Language	Junior_Senior	0.06	308	57.47

Independent sample ID	Publication year	SRL Phases	SRL strategies	Discipline	Educational stage	Correlation	Sample size	Percentage of female
Zhang, F & Liu, C_5	2012	Performance	Metacognitive_monitoring	Integrated	Junior_Senior	0.22	308	57.47
Zhang, F & Liu, C_6	2012	Reflection	Self-evaluation	Language	Junior_Senior	0.13	308	57.47
Zhang, F & Liu, C_7	2012	Reflection	Self-evaluation	Science	Junior_Senior	0.14	308	57.47
Zhang, F & Liu, C_8	2012	Reflection	Self-evaluation	Language	Junior_Senior	0.01	308	57.47
Zhang, F & Liu, C_9	2012	Reflection	Self-evaluation	Integrated	Junior_Senior	0.13	308	57.47
Zhang, F & Liu, C_10	2012	Reflection	Attention_focusing	Language	Junior_Senior	0.13	308	57.47
Zhang, F & Liu, C_11	2012	Performance	Metacognitive_monitoring	Language	Junior_Senior	0.2	308	57.47
Zhang, F & Liu, C_12	2012	Performance	Attention_focusing	Science	Junior_Senior	0.11	308	57.47
Zhang, F & Liu, C_13	2012	Performance	Attention_focusing	Language	Junior_Senior	0.01	308	57.47
Zhang, F & Liu, C_14	2012	Performance	Attention_focusing	Integrated	Junior_Senior	0.12	308	57.47
Zhang, F & Liu, C_15	2012	Performance	Task_strategies	Language	Junior_Senior	0.13	308	57.47
Zhang, F & Liu, C_16	2012	Performance	Task_strategies	Science	Junior_Senior	0.12	308	57.47
Zhang, F & Liu, C_17	2012	Performance	Task_strategies	Language	Junior_Senior	-0.02	308	57.47
Zhang, F & Liu, C_18	2012	Performance	Metacognitive_monitoring	Science	Junior_Senior	0.23	308	57.47
Zhang, F & Liu, C_19	2012	Performance	Task_strategies	Integrated	Junior_Senior	0.12	308	57.47
Zhang, F & Liu, C_20	2012	Forethought	Goal_setting	Language	Junior_Senior	0.17	308	57.47
Zhang, F & Liu, C_21	2012	Forethought	Goal_setting	Science	Junior_Senior	0.31	308	57.47
Zhang, F & Liu, C_22	2012	Forethought	Goal_setting	Language	Junior_Senior	0.16	308	57.47
Zhang, F & Liu, C_23	2012	Forethought	Goal_setting	Integrated	Junior_Senior	0.28	308	57.47
Zhang, F & Liu, C_24	2012	Performance	Metacognitive_monitoring	Language	Junior_Senior	0.06	308	57.47
Zhang, F & Liu, C_25	2012	Performance	Metacognitive_monitoring	Language	Junior_Senior	0.19	308	57.47
Zhang, F & Liu, C_26	2012	Performance	Metacognitive_monitoring	Science	Junior_Senior	0.23	308	57.47
Zhang, F & Liu, C_27	2012	Performance	Metacognitive_monitoring	Language	Junior_Senior	0.07	308	57.47
Zhang, F & Liu, C_28	2012	Performance	Metacognitive_monitoring	Integrated	Junior_Senior	0.23	308	57.47
Zhang, J. Liu, J. W. & Wu, Q. L_1	2012	Performance	Metacognitive_monitoring	Integrated	Junior	0.07	600	51.67
Zhang, J. Liu, J. W. & Wu, Q. L_2	2012	Forethought	Self-efficacy	Integrated	Junior	0.13	600	51.67
Zhang, J. Liu, J. W. & Wu, Q. L_3	2012	Forethought	Goal_orientation	Integrated	Junior	-0.08	600	51.67
Zhang, J. Liu, J. W. & Wu, Q. L_4	2012	Forethought	Goal_orientation	Integrated	Junior	-0.03	600	51.67
Zhang, J. Liu, J. W. & Wu, Q.	2012	Forethought	Goal_orientation	Integrated	Junior	-0.01	600	51.67

Independent sample ID	Publication year	SRL Phases	SRL strategies	Discipline	Educational stage	Correlation	Sample size	Percentage of female
L_5								
Zhang, J. Liu, J. W. & Wu, Q. L_6	2012	Forethought	Goal_orientation	Integrated	Junior	0.14	600	51.67
Zhang, J. X & Zhang, C_1	2008	Performance	Metacognitive_monitoring	Integrated	Elementary_Junior	0.125	674	NA
Zhang, J. X & Zhang, C_2	2008	Performance	Metacognitive_monitoring	Integrated	Elementary_Junior	0.147	674	NA
Zhang, J. X & Zhang, C_3	2008	Performance	Metacognitive_monitoring	Language	Elementary_Junior	0.074	674	NA
Zhang, J_1	2011	Forethought	Self-efficacy	Language	Senior	0.148	523	52.58
Zhang, J_2	2011	Forethought	Self-efficacy	Language	Senior	0.228	523	52.58
Zhang, J_3	2011	Performance	Task_strategies	Language	Senior	0.076	523	52.58
Zhang, J_4	2011	Performance	Task_strategies	Language	Senior	0.079	523	52.58
Zhang, J_5	2011	Performance	Task_strategies	Language	Senior	0.163	523	52.58
Zhang, J_6	2011	Performance	Task_strategies	Language	Senior	0.197	523	52.58
Zhang, J_7	2011	Performance	Task_strategies	Language	Senior	0.179	523	52.58
Zhang, J_8	2011	Performance	Task_strategies	Language	Senior	0.2	523	52.58
Zhang, L & You, Z. Q	2014	Performance	Task_strategies	Integrated	Junior	0.45	448	47.1
Zhang, L. H, Sun, X. J, & Feng, J. Y	2011	Performance	Metacognitive_monitoring	Language	Elementary	0.596	60	55
Zhang, W	2013	Forethought	Self-efficacy	Science	Junior	0.4	84	40.48
Zhang, W, H & Shen, J. L_1	2006	Forethought	Goal_orientation	Language	Elementary	-0.03	686	49.85
Zhang, W, H & Shen, J. L_2	2006	Forethought	Goal_orientation	Language	Elementary	0.11	686	49.85
Zhang, W, H & Shen, J. L_3	2006	Forethought	Goal_orientation	Science	Elementary	-0.09	686	49.85
Zhang, W, H & Shen, J. L_4	2006	Forethought	Goal_orientation	Science	Elementary	0.1	686	49.85
Zhang, W, H & Shen, J. L_5	2006	Forethought	Goal_orientation	Language	Elementary	-0.1	686	49.85
Zhang, W, H & Shen, J. L_6	2006	Forethought	Goal_orientation	Language	Elementary	0.15	686	49.85
Zhang, W, H & Shen, J. L_7	2006	Forethought	Goal_orientation	Integrated	Elementary	-0.09	686	49.85
Zhang, W, H & Shen, J. L_8	2006	Forethought	Goal_orientation	Integrated	Elementary	0.15	686	49.85
Zhang, X. H & Gao, C. Y_1	2010	Performance	Task_strategies	Language	Senior	0.194	200	34.5
Zhang, X. H & Gao, C. Y_2	2010	Performance	Metacognitive_monitoring	Language	Senior	0.081	200	34.5
Zhang, X. H & Gao, C. Y_3	2010	Performance	Metacognitive_monitoring	Language	Senior	0.231	200	34.5
Zhang, X. H & Gao, C. Y_4	2010	Performance	Metacognitive_monitoring	Language	Senior	0.155	200	34.5
Zhang, X. H & Gao, C. Y_5	2010	Performance	Metacognitive_monitoring	Language	Senior	0.083	200	34.5
Zhang, X. X & Yang, Y. X_1	2012	Forethought	Goal_orientation	Language	Junior	-0.103	72	61.11

Independent sample ID	Publication year	SRL Phases	SRL strategies	Discipline	Educational stage	Correlation	Sample size	Percentage of female
Zhang, X. X & Yang, Y. X_2	2012	Forethought	Goal_orientation	Language	Junior	-0.012	72	61.11
Zhang, X. X & Yang, Y. X_3	2012	Forethought	Goal_orientation	Language	Junior	-0.516	72	61.11
Zhang, X. X & Yang, Y. X_4	2012	Forethought	Goal_orientation	Language	Junior	0.046	72	61.11
Zhang,Y & Li,Q.W_1	2010	Forethought	Goal_orientation	Integrated	Junior	0.162	1140	NA
Zhang,Y & Li,Q.W_2	2010	Forethought	Goal_orientation	Integrated	Junior	0.251	1140	NA
Zhang,Y & Li,Q.W_3	2010	Reflection	Attribution	Integrated	Junior	-0.158	1140	NA
Zhang,Y.F, Wei, X.C, & He,H_1	2014	Reflection	Attribution	Language	Senior	0.041	296	63.18
Zhang,Y.F, Wei, X.C, & He,H_2	2014	Reflection	Attribution	Science	Senior	0.081	296	63.18
Zhou,L.H & Liu, Y_1	2012	Performance	Attention_focusing	Science	Senior	0.098	305	39.67
Zhou,L.H & Liu, Y_2	2012	Reflection	Self-satisfaction/affect	Science	Senior	0.082	305	39.67
Zhou,L.H & Liu, Y_3	2012	Reflection	Self-evaluation	Science	Senior	0.348	305	39.67
Zhou,L.H & Liu, Y_4	2012	Forethought	Task_interest/value	Science	Senior	0.137	305	39.67
Zhou,L.H & Liu, Y_5	2012	Forethought	Task_interest/value	Science	Senior	0.219	305	39.67
Zhou,L.H & Liu, Y_6	2012	Reflection	Self-evaluation	Science	Senior	0.348	305	39

Intervention Studies								
Chen, X. Q., Zhang, Y. X., Jiang, Q., & Meng, X, Q	2014	Performance	Task_strategies	Language	Senior	NA	111	NA
Gao, R. C., Liu, R. D	2011	Pooled	Integrated	Integrated	Junior	NA	184	44
Jiang, B., & Tan, D. L_1	2007	Performance	Task_strategies	Language	Elementary	NA	47	NA
Jiang, B., & Tan, D. L_2	2007	Performance	Task_strategies	Science	Elementary	NA	47	NA
Xu, J. B	2012	Pooled	Integrated	Language	Junior	NA	98	NA

Note. This table provides the details for each independent sample of the studies included in the meta-analysis. *Independent sample ID* identifies the independent samples from each study. *Publication year* lists the year of publication. *SRL phases* classifies the strategies studied in each sample into “forethought”, “performance”, or “self-reflection” phases according to Zimmerman’s (2008) cyclical model of SRL; “pooled” indicates the case that two or more phases were involved. *SRL strategies* indicates the major self-regulated learning strategies studies in each sample; “integrated” indicates the case that two or more strategies were studied. *Discipline* refers to the category of academic disciplines, including language (Chinese and English), science performance (mathematics and physics), and integrated (i.e., when the study did not specify disciplines but use integrated interim or final grades). *Educational stage* refers to the level of students in the sample, including elementary, junior high, and senior high schools. *Correlation* records the correlation coefficient between SRL strategies and academic performances in the sample. *Sample size* records the number of participants in the sample. *Percentage of female* records the proportion of female students in the sample.

Table A.2 Treatment fidelity information of four intervention studies.

Independent sample ID	Chen et al.	Gao & Liu	Jiang & Tan_1	Jiang & Tan_2	Xu
Publication year	2014	2011	2007	2007	2012
SRL Phases	Performance	Pooled	Performance	Performance	Pooled
Educational stage	Senior	Junior	Elementary	Elementary	Junior
Discipline	Language	Integrated	Language	Science	Language
Sample size	111	184	47	47	98
Treated Mean	88.732	2.24	92.35	88.23	78.925
Treated SD	4.731	0.61	3.71	6.46	13.75
Treated sample size	56	49	26	26	49
Control Mean	87.759	1.62	92.24	84.05	73.836
Control SD	8.321	0.61	2.76	5.44	12.912
Control sample size	55	135	21	21	49
Training of instructors	No	No	Yes	Yes	No
Protocol	No	Yes	Yes	Yes	Yes
Pre-post measures	Yes	No	Yes	Yes	Yes
Number of groups	2	2	2	2	2
Recording of sessions	Yes	Yes	Yes	Yes	Yes
Number of sessions	10	5	12	12	20
Interview check	Yes	No	No	No	No

Note. This table provides the designs for each independent sample from the intervention studies included in the meta-analysis. *Publication year* lists the year of publication. *SRL phases* classifies the strategies studied in each sample into "forethought", "performance", or "self-reflection" phases according to Zimmerman's (2008) cyclical model of SRL; "pooled" indicates the case that two or more phases were involved. *Discipline* refers to the category of academic disciplines, including language (Chinese and English), science performance (mathematics and physics), and integrated (i.e., when the study did not specify disciplines but use integrated interim or final grades). *Educational stage* refers to the level of students in the sample, including elementary, junior high, and senior high schools. *Treated mean* through *control sample size* respectively records the mean, standard deviation (SD), and sample size in the treatment group and control group. *Training of instructors* through *interview check* indicates the fidelity information in the sample. *Training of instructors* refers to whether or not the researcher trained the instructors before the intervention. *Protocol* refers to whether or not the written protocol for intervention is available to the instructors. *Pre-post measures* indicates whether or not the academic performance was measured before and after the intervention. *Number of groups* records the number of student groups in the intervention. *Recording of sessions* refers to whether or not the intervention was recorded. *Number of sessions* records the number of intervention sessions during the study. *Interview check* refers to whether or not the researchers interviewed the students after the intervention to ensure the effectiveness of the intervention.

References included in the meta-analysis

An, M., & Yu, J.Y. (2009). The relationship between metacognitive monitoring and academic performance of secondary school students. *Inner Mongolia Education, 2009*(24), 11-13. Retrieved from http://kns.cnki.net/KCMS/detail/detail.aspx?dbcode=CFJD&dbname=CJFDN0911&filename=LMJY200924005&v=MTU4NzkxRnJDVVJMS2VaK1pzRnkzbVY3M0FLU0RCZDdHNEh0ak9xNDlGWVlSOGVYMUx1eFlTN0RoMVQzcVRyV00=

Cao, L. R., & Cao, X. H. (2004). The influence of time management disposition, cognitive style, and meta-worry on the high school students' achievement. *Chinese Journal of Ergonomics, 10(3),* 13-15. doi: 10.13837/j.issn.1006-8309.2004.03.005

Chang, H. Q., & Pan, Y. (2013). A correlational study of time management and academic performance in secondary vocational school students. *Academy, 2013(22),* 175-176. Retrieved from http://kns.cnki.net/KCMS/detail/detail.aspx?dbcode=CFJD&dbname=CJFDN1214&filename=XYJK201322133&v=MjMxMjFYMUx1eFlTN0RoMVQzcVRyV00xRnJDVVJMS2VaK1pzRnkzbVVMM05QVFRCWmJHNEg5TE9yWTVHWjRSOGU=

Chen, C., Gong, S. Y., & Chai, X. Y. (2013). Parent goals, classroom goal structures, personal achievement goal orientations and academic performance for middle school students. *Psychological Research, 6(2),* 78-84. Retrieved from http://kns.cnki.net/KCMS/detail/detail.aspx?dbcode=CJFQ&dbname=CJFD2013&filename=OXLY201302015&v=MTU3Mjk5TE1yWTlFWVlSOGVYMUx1eFlTN0RoMVQzcVRyV00xRnJDVVJMS2VaK1pzRnkzbVU3L1BLalhIZDdHNEg=

Chen, J. (2009). The relationship between goal orientation, seeking help and academic performance of high school students. *Shanghai Youth, 2009(1),* 21-23. Retrieved from http://kns.cnki.net/KCMS/detail/detail.aspx?dbcode=CJFQ&dbname=CJFD2009&filename=QNGL200901013&v=MDE0MzlPTkNQTVlyRzRIdGpNcm85RVo0UjhlWDFMdXhZUzdEaDFUM3FUcldNMUZyQ1VSTEtlWitac0Z5M2xXN3o=

Chen, J. J., & Li, S. F. (2012). The paths of junior school students' achievement attribution and academic emotions forecasting their academic achievement. *Chinese Journal of Clinical Psychology, 20(3),* 392-394. doi: 10.16128/j.cnki.1005-3611.2012.03.004

Chen, L. J. (2011). Relationship among achievement goal orientation, self-efficacy and academic performance of high school students. *China Journal of Health Psychology, 19(6),* 718-719. Retrieved from http://kns.cnki.net/KCMS/detail/detail.aspx?dbcode=CJFQ&dbname=CJFD2011&filename=JKXL201106039&v=MTU0MjhIOURNcVk5R2JZUjhlWDFMdXhZUzdEaDFUM3FUcldNMUZyQ1VSTEtlWitac0Z5M2xVTDNBTHliVFlyRzQ=

Chen, M. G., & Hu, Z. B. (2008). Research on the relationships among goal orientation, learning strategy, attribution and academic performance in high school students. *Psychological Exploration, 28(3),* 58-62. Retrieved from http://kns.cnki.net/KCMS/detail/detail.aspx?dbcode=CJFQ&dbname=CJFD2008&filename=XLXT200803014&v=Mjk1MzhSOGVYMUx1eFlTN0RoMVQzcVRyV00xRnJDVVJMS2VaK1pzRnkzbFZMM0JQU0hUZXJHNEh0bk1ySTlFWUk=

Chen, X. Q., Zhang, Y. X., Jiang, Q., & Meng, X, Q. (2014). The effects of metacognitive strategy training on English learning in high school students. *Middle school students, 2014(11),* 109-110. Retrieved from http://kns.cnki.net/KCMS/detail/detail.aspx?dbcode=CFJD&dbname=CJFDLASN2014&filename=YGZB201431089&v=MDA4ODNVUkxLZVorWnNGeTNsVXJyUFBDclJiTEc0SDlYUHJvOU5iWVI4ZVgxTHV4WVM3RGgxVDNxVHJXTTFGckM=

Chi, Y. K., & Zhou, G. T. (2003). The effects of perceived pedagogical caring and self-regulate learning on academic achievement. *Journal of Guangdong Education Institute, 23(4),* 47-51. Retrieved from http://kns.cnki.net/KCMS/detail/detail.aspx?dbcode=CJFQ&dbname=CJFD2003&filename=GJXY200304010&v=MTY1NjJmVGQ3RzRIdExNcTQ5RVpJUjhlWDFMdXhZUzdEaDFUM3FUcldNMUZyQ1VSTEtlWitac0Z5M2tWYnpQSWk=

Cui, N., Liu, Y. Y., & Gao, B. Y. (2008). The relationship between achievement goal, examining anxiety and performance in senior students. *China of Journal of Health Psychology, 16(4),* 436-438. Retrieved from http://kns.cnki.net/KCMS/detail/detail.aspx?dbcode=CJFQ&dbname=CJFD2008&filename=JKXL200804036&v=MTY0NzkxRnJDVVJMS2VaK1pzRnkza1ZMelBMeWJUWXJHNEh0bk1xNDlHWW9SOGVYMUx1eFlTN0RoMVQzcVRyV00=

Gao, R. C., Liu, R. D., Wang, D., He, M. J., & Yuan, Z. (2011). Types of self-regulated learning of junior middle school students and their influence on learning. *Psychological Development and Education, 2011(1),* 76-82. Retrieved from http://kns.cnki.net/KCMS/detail/detail.aspx?dbcode=CJFQ&dbname=CJFD2011&filename=XLFZ201101011&v=MTgxOTBETXJvOUVaWVI4ZVgxTHV4WVM3RGgxVDNxVHJXTTFGckNVUkxLZVorWnNGeTNrVnJ6UFBTSE5kTEc0SDk=

Ge, M, G., & Jin, Y. (2005). A study on the relationships between English learning strategies and academic achievement of middle school students. *Psychological Science, 28(2),* 451-453. Retrieved from http://kns.cnki.net/KCMS/detail/detail.aspx?dbcode=CJFQ&dbname=CJFD2005&filename=XLKX200502049&v=MzI2MTVUcldNMUZyQ1VSTEtlWitac0Z5cmhWNzNCUFNIQWRyRzRIdFRNclk5QmJZUjhlWDFMdXhZUzdEaDFUM3E=

Gu, S. H., Xin, T., & Li, H. (1998). A correlational study of attribution, learning strategy, and academic achievement in junior high school students. *Psychological Development and Education, 1998(2),* 21-25. Retrieved from http://kns.cnki.net/KCMS/detail/detail.aspx?dbcode=CJFQ&dbname=CJFD9899&filename=XLFZ802.004&v=MDYxMjFTSE5kTHU0SE0vTXI0c3FGNTRPZmdnNXpoQVU0amg0T1g2VHJIMDNlYk9jUkxxZlpPZHNFeTNrVUE9PVA=

Guo, W. (2008). A study on the relationship between the factors of personality and academic results of middle school students. *Journal of Fu Qing Branch of Fujian Normal University, 2008(11),* 46-50. Retrieved from http://kns.cnki.net/KCMS/detail/detail.aspx?dbcode=CJFQ&dbname=CJFD2008&filename=FJFQ200806015&v=MjAzMjFyQ1VSTEtlWitac0Z5cmdWN3pCSXlmTmY3RzRIdG5NcVk5RVlZUjhlWDFMdXhZUzdEaDFUM3FUcldNMUY=

Han, X. H., Zheng, L. N., & Liu, R. J. (2015). The mediation effect of self-regulated learning in parent involvement and academic achievement in higher elementary school students. *Journal of Liaocheng University, 28(1),* 74-78. Retrieved from http://kns.cnki.net/KCMS/detail/detail.aspx?dbcode=CJFQ&dbname=CJFDLAST2015&filename=TALK20

1501016&v=MDYzMjlEaDFUM3FUcldNMUZyQ1VSTEtlWitac0Z5cmdVTDNQTVN6SFpiRzRIOVRNc m85RVlvUjhlWDFMdXhZUzc=

Ji, H. Y. (2005). The relationship between middle school students' self-regulated learning development and academic performance. *Journal of Chuzhou University, 7(5),* 97-100. Retrieved from http://kns.cnki.net/KCMS/detail/detail.aspx?dbcode=CJFQ&dbname=CJFD2005&filename=CZSB200505029&v=MDc5MjJmWWJMRzRIdFRNcW85SGJZUjhlWDFMdXhZUzdEaDFUM3FUcldNMUZyQ1VSTEtlWitac0Z5cm5XN3ZKSmo=

Jiang, M. R., Song, H. S., & Zhang, L. (2014). Research on correlation between self-evaluation ability and academic achievement. *Journal of Kashgar Teachers College, 35(3),* 66-68. Retrieved from http://kns.cnki.net/KCMS/detail/detail.aspx?dbcode=CJFQ&dbname=CJFD2014&filename=KSSB201403020&v=MDEwODI3WWJMRzRIOVhNckk5SFpJUjhlWDFMdXhZUzdEaDFUM3FUcldNMUZyQ1VSTEtlWitac0Z5cm5VNzdLTGo=

Jiang, Y. Z., & Zhang, H. Z. (2012). A correlational research of self-esteem, self-efficacy and academic performance in junior high school students. *Educational measurement and evaluation, 2012(4),* 43-50. Retrieved from http://kns.cnki.net/KCMS/detail/detail.aspx?dbcode=CJFQ&dbname=CJFD2012&filename=PJYC201204013&v=MDI1ODk5UE1xNDlFWjRSOGVYMUx1eFlTN0RoMVQzcVRyV00xRnJDVVJMS2VaK1pzRnlyblVMM0xOU2ZTYmJHNEg=

Jiang, B., & Tan, D. L. (2007). The effects of peer learning on academic performance and social development of junior high school students. *Journal of Tianjin Academy of Educational Science, 2007(1),* 33-35. Retrieved from http://kns.cnki.net/KCMS/detail/detail.aspx?dbcode=CJFQ&dbname=CJFD2007&filename=TJJK200701008&v=MDM5NjViRzRIdGJNcm85RmJJUjhlWDFMdXhZUzdEaDFUM3FUcldNMUZyQ1VSTEtlWitac0Z5cm5WNzNBTVNmQlo=

Jiang, J. C., & Liu, H. S. (2005). Research on the relationship between the achievement goal orientation and the learning strategy & the academic performance. *Psychological Development and Education, 2005(2),* 56-61. Retrieved from http://kns.cnki.net/KCMS/detail/detail.aspx?dbcode=CJFQ&dbname=CJFD2005&filename=XLFZ200502011&v=MDQyMjg3RGgxVDNxVHJXTTFGckNVUkxLZVorWnNGeXJtV3IzSlBTSE5kTEc0SHRUTXJZOUVaWVI4ZVgxTHV4WVM=

Jiao, C. Z. (2008). Relationship of mathematic interest self-efficacy and mathematic achievement of junior school students. *Journal of Mathematics Education, 17(2),* 44-46. Retrieved from http://kns.cnki.net/KCMS/detail/detail.aspx?dbcode=CJFQ&dbname=CJFDN0508&filename=SXYB200802011&v=MjI2NjBGckNVUkxLZVorWnNGeXJtVkw3Tk5qWFNiTEc0SHRuTXJZOUVaWVI4ZVgxTHV4WVM3RGgxVDNxVHJXTTE=

Jin, G. Y., Gao, J. J., & Chen, Y, W. (2012). How learning strategy and self-efficacy predict achievement in different situations. *Journal of Zhejiang University, 39(2),* 231-238. Retrieved from http://kns.cnki.net/KCMS/detail/detail.aspx?dbcode=CJFQ&dbname=CJFD2012&filename=HZDX201202023&v=MTY2ODc0SDlQTXJZOUhaNFI4ZVgxTHV4WVM3RGgxVDNxVHJXTTFGckNVUkxLZVorWnNGeXJtVTd2QkxUZlBkckc=

Lei, L., Wang, L., & Culjak, T. (2001). The role of goal-orientation in self-regulated learning. *Acta Psychologica Sinica, 33(4),* 349-353. Retrieved from http://kns.cnki.net/KCMS/detail/detail.aspx?dbcode=CJFQ&dbname=CJFD2001&filename=XLXB200104010&v=MjI5OTFNcTQ5RVpJUjhlWDFMdXhZUzdEaDFUM3FUcldNMUZyQ1VSTEtlWitac0Z5cm1Vci9LUFNIVGJMRzRIdEQ=

Li, B., & Yang, J. L. (2004). Relations of time management disposition, self-efficiency, attribution of junior school students to academic achievement. *Psychological Exploration, 24(4),* 67-71. Retrieved from http://kns.cnki.net/KCMS/detail/detail.aspx?dbcode=CJFQ&dbname=CJFD2004&filename=XLXT200404016&v=MjA4NzVsVmJyQlBTSFRlckc0SHRYTXE0OUVZb1I4ZVgxTHV4WVM3RGgxVDNxVHJXTTFGckNVUkxLZVorWnNGeXI=

Li, Z. X., & Hu, Y. R. (2014). The relationship between goal orientation and academic achievement in junior 初 high school students: The mediation of self-efficacy. *Journal of Hubei University of Science and Technology, 34(9),* 123-125. Retrieved from http://kns.cnki.net/KCMS/detail/detail.aspx?dbcode=CJFQ&dbname=CJFDLAST2015&filename=XNSZ201409053&v=MTc1MTh2QVBTUFlkTEc0SDlYTXBvOUFaNFI4ZVgxTHV4WVM3RGgxVDNxVHJXTTFGckNVUkxLZVorWnNGeXJnVmI=

Li, H. X., Lin, X., & Si, J. W. (2015). The relationship between time management disposition and academic achievement in boarding primary school students: The mediating role of self-regulate learning. *Psychological Research, 8(6),* 90-96. Retrieved from http://kns.cnki.net/KCMS/detail/detail.aspx?dbcode=CJFQ&dbname=CJFDLAST2016&filename=OXLY201506015&v=MTc3MzdXTTFGckNVUkxLZVorWnNGeXJsVjcvTEtqWEhkN0c0SDlUTXFZOUVZWVI4ZVgxTHV4WVM3RGgxVDNxVHI=

Li, J. D., An, M., He, T. T., & Ge, G. Y. (2009). The relationships among psychological resilience, meta-cognition, self-efficacy, test anxiety and academic performance in middle school students. *Examination Weekly, 2009(49),* 13-14. Retrieved from http://kns.cnki.net/KCMS/detail/detail.aspx?dbcode=CFJD&dbname=CJFDN0911&filename=KDZK200949010&v=MTE5ODFyQ1VSTEtlWitac0Z5cmxVNy9MTGluUlpiRzRIdGpJcG85RVpJUjhlWDFMdXhZUzdEaDFUM3FUcldNMUY=

Li, J., Zhang, J., & Zhu, L. Q. (2011). The development of middle school students' self-evaluation and its relationship with academic achievement. *Psychological Science, 34(3),* 619-624. Retrieved from http://kns.cnki.net/KCMS/detail/detail.aspx?dbcode=CJFQ&dbname=CJFD2011&filename=XLKX201103020&v=MjcxMzQ5SFpJUjhlWDFMdXhZUzdEaDFUM3FUcldNMUZyQ1VSTEtlWitac0Z5cmtWYnZQUFNIQWRyRzRIOURNckk=

Li, S., & Yu, X. X. (2002). The effects of self-concept, self-monitoring and learning strategy on academic achievement in middle school students. *Journal of Ningbo University, 24(3),* 18-22. Retrieved from http://kns.cnki.net/KCMS/detail/detail.aspx?dbcode=CJFQ&dbname=CJFD2002&filename=LBJY200203004&v=MjU1MjdTN0RoMVQzcVRyV00xRnJDVVJMS2VaK1pzRnlya1Y3ekJLUy9CZDdHNEh0UE1ySTlGWUlSOGVYMUx1eFk=

Li, X. Y., & Li, J. W. (2004). Relative study on middle school students' mathematical achievement motivation self-regulated learning and mathematical achievement. *Journal of Mathematics Education, 13(2),* 52-54. Retrieved from

http://kns.cnki.net/KCMS/detail/detail.aspx?dbcode=CFJD&dbname=CJFDN7904&filename=SXYB200402018&v=MTM2NzN5cmtVYnJQTmpYU2JMRzRIdFhNclk5RWJJUjhlWDFMdXhZUzdEaDFUM3FUcldNMUZyQ1VSTEtlWitac0Y=

Liu, J. X., Xin, T., Huang, G. Q., & Shen, J. L. (2000). A correlational study of learning motivation, learning 中 strategy and academic performance in middle school students. *Theory and Practice of Education, 20(9),* 54-58. Retrieved from http://kns.cnki.net/KCMS/detail/detail.aspx?dbcode=CJFD&dbname=CJFD2000&filename=JYLL200009011&v=MjY5MDg3RGgxVDNxVHJXTTFGckNVUkxLZVorWnNGeXJrVTd6TUx6VEhZckc0SHRITXBvOUVaWVI4ZVgxTHV4WVM=

Liu, J. L., & Liu, S. X. (2016). The relationship between learning attitude and academic achievement of junior middle school students. *Journal of Gannan Normal University, 2016(2),* 106-109. Retrieved from http://kns.cnki.net/KCMS/detail/detail.aspx?dbcode=CJFQ&dbname=CJFDLAST2016&filename=GNSY201602029&v=MjU1NTc3N0xJaVBZZDdHNEg5Zk1yWTlIYllSOGVYMUx1eFlTN0RoMVQzcVRyV00xRnJDVVJMS2VaK1pzRnl2aFc=

Liu, T. (2015). On relationship between academic adaptability, academic self-efficacy and academic performance of junior middle school students. *Journal of Baicheng Normal University, 29(6),* 62-66. Retrieved from http://kns.cnki.net/KCMS/detail/detail.aspx?dbcode=CJFQ&dbname=CJFDLAST2015&filename=BCSF201506018&v=MTc1NzFzRnl2aFY3dktKeTdZYUxHNEg5VE1xWTlFYklSOGVYMUx1eFlTN0RoMVQzcVRyV00xRnJDVVJMS2VaK1o=

Ran, R., Huang, Y. Q., & Yu, J. (2012). The relationship between self-efficacy and academic achievement of junior high school students in the mainland Tibetan class. *Journal of Jiangsu Teachers University of Technology, 18(1),* 79-83. Retrieved from http://kns.cnki.net/KCMS/detail/detail.aspx?dbcode=CJFQ&dbname=CJFD2012&filename=CZJF201201020&v=MTY5ODZHNEg5UE1ybzlIWklSOGVYMUx1eFlTN0RoMVQzcVRyV00xRnJDVVJMS2VaK1pzRnl2aFVickpKamZCYUw=

Wang, J. X., & Zhang, K. (2008). Attribution, self-efficacy, examination anxiety and performance of middle school student. *Psychological Research, 1(1),* 89-91. Retrieved from http://kns.cnki.net/KCMS/detail/detail.aspx?dbcode=CJFQ&dbname=CJFD2008&filename=OXLY200801019&v=MDM3MTc3dk9LalhIZDdHNEh0bk1ybzlFYllSOGVYMUx1eFlTN0RoMVQzcVRyV00xRnJDVVJMS2VaK1pzRnl2aFU=

Wang, L. J. (2013). Research on the correlation among middle school students’ cognitive interference, achievement goal orientation and their academic performance. *Journal of Zhongzhou University, 30(5),* 98-104. Retrieved from http://kns.cnki.net/KCMS/detail/detail.aspx?dbcode=CJFQ&dbname=CJFDHIS2&filename=ZOZO201305023&v=MjcwNDNVUkxLZVorWnNGeXZoVXJySVB5TFJZYkc0SDlMTXFvOUhaNFI4ZVgxTHV4WVM3RGgxVDNxVHJXTTFGckM=

Wang, Y. X., Cao, Y. T., & Tang, X. L. (2013). Senior middle school students’ academic self-efficacy and related study of its relationship with academic achievements. *Journal of Weinan Normal University, 28(1),* 141-144. Retrieved from http://kns.cnki.net/KCMS/detail/detail.aspx?dbcode=CJFD&dbname=CJFD2013&filename=WOLF201301032&v=MDY0MDNVUkxLZVorWnNGeXZnVTd2TU1pTEhhTEc0SDlMTXJvOUdab1I4ZVgxTHV4WV

M3RGgxVDNxVHJXTTFGckM=

Wang, Y. Z., Wang, S. Z., & Ou, Y. L. (2005). A study on the relationships of academic achievements, academic self-efficacy and intrinsic motivations among middle school students in poverty-stricken areas. *Psychological Science, 28(4),* 826-829. Retrieved from http://kns.cnki.net/KCMS/detail/detail.aspx?dbcode=CJFQ&dbname=CJFD2005&filename=XLKX200504013&v=MTcxOTZyV00xRnJDVVJMS2VaK1pzRnl2Z1Y3L0FQU0hBZHJHNEh0VE1xNDlFWjRSOGVYMUx1eFlTN0RoMVQzcVQ=

Wei, X. C., & Zhang, Y. F. (2014). A correlational study on the relationships among self-efficacy, attribution, and Chinese academic performance in middle school students. *Research in Teaching, 37(4),* 117-120. Retrieved from http://kns.cnki.net/KCMS/detail/detail.aspx?dbcode=CJFQ&dbname=CJFD2014&filename=JIYJ201404032&v=Mjc5MDJUU1pMRzRIOVhNcTQ5R1pvUjhlWDFMdXhZUzdEaDFUM3FUcldNMUZyQ1VSTEtlWitac0Z5dm5XNy9MTHk=

Wu, J. Y., Hu, X. L., & Peng, J. S. (2011). Relationship between self-efficacy and academic performance of physics of senior middle school students. *Education and Teaching Research, 25(2),* 122-125. Retrieved from http://kns.cnki.net/KCMS/detail/detail.aspx?dbcode=CFJD&dbname=CJFDN0911&filename=WUJS201106002&v=MDQyMTFNcVk5RlpvUjhlWDFMdXhZUzdEaDFUM3FUcldNMUZyQ1VSTEtlWitac0Z5dm5WYnpJTWpqQmZiRzRIOUQ=

Xu, J. B. (2012). An experimental study of self-regulated learning in English reading education. *English Teaching Research and Practice in Primary and Secondary School, 2012(3),* 80-83. Retrieved from http://kns.cnki.net/KCMS/detail/detail.aspx?dbcode=CFJD&dbname=CJFDN1214&filename=YYXS201203026&v=MDI0NTVuVXJ2QVBEVFRmYkc0SDlQTXJJOUhZb1I4ZVgxTHV4WVM3RGgxVDNxVHJXTTFGckNVUkxLZVorWnNGeXY=

Xu, L. L, Guo, W. (2007). The relationships among goal orientation, English learning strategies and academic performance in senior high school students. *Data of Culture and Education, 2007(3),* 146-147. Retrieved from http://kns.cnki.net/KCMS/detail/detail.aspx?dbcode=CFJD&dbname=CJFDN0508&filename=WJZZ200703092&v=MTg0ODZHNEh0Yk1ySTlNWm9SOGVYMUx1eFlTN0RoMVQzcVRyV00xRnJDVVJMS2VaK1pzRnl2bVVidkJNaWZSZEw=

Yao, C. L. (2013). A correlational research of English learning anxiety, meta-cognition and academic performance. *Teaching Practice, 2013(2),* 54-60. Retrieved from http://kns.cnki.net/KCMS/detail/detail.aspx?dbcode=CFJD&dbname=CJFDLASN2014&filename=XKJX201302014&v=MDg1MzQ5RVlJUjhlWDFMdXhZUzdEaDFUM3FUcldNMUZyQ1VSTEtlWitac0Z5dm1VTDNBUFNiQmRyRzRIOUxNclk=

Yao, D. W., Yan, L. F., Musa, A. & Liu, G. (2011). Relationship among cognitive styles, learning motivation and strategies of junior students and the effect to school work achievement. *Psychological Research, 4(6),* 92-96. Retrieved from http://kns.cnki.net/KCMS/detail/detail.aspx?dbcode=CJFQ&dbname=CJFD2011&filename=OXLY201106019&v=MjExNDIxTHV4WVM3RGgxVDNxVHJXTTFGckNVUkxLZVorWnNGeXZsVzd2T0tqWEhkN0c0SDlETXFZOUViWVI4ZVg=

Yuan, L. X. (2005). The study of relationship between achievement goals, self-efficacy, learning strategies and academic achievement. *Journal of Guangdong Education Institute, 25(6),* 58-61. Retrieved from http://kns.cnki.net/KCMS/detail/detail.aspx?dbcode=CJFQ&dbname=CJFD2005&filename=GJXY200506013&v=MTc0OTVUcldNMUZyQ1VSTEtlWitac0Z5dmxWYnpQSWlmVGQ3RzRIdFRNcVk5RVo0UjhlWDFMdXhZUzdEaDFUM3E=

Zhang, F., & Liu, C. (2012). Self-control of time management and academic performance of middle school students. *Psychological Research, 5(4),* 79-84. Retrieved from http://kns.cnki.net/KCMS/detail/detail.aspx?dbcode=CJFQ&dbname=CJFD2012&filename=OXLY201204015&v=MTA4MTVFWVlSOGVYMUx1eFlTN0RoMVQzcVRyV00xRnJDVVJMS2VaK1pzRnl2a1Zidk1LalhIZDdHNEg5UE1xNDk=

Zhang, J. (2011). The relationships among English learning strategies, self-efficacy, and academic performance in senior high school students. *Journal of Basic English Education, 13(2),* 42-47. Retrieved from http://kns.cnki.net/KCMS/detail/detail.aspx?dbcode=CJFQ&dbname=CJFD2011&filename=SDWG201102011&v=MjIwNzJGeXZrVUx2QU5pbmNhYkc0SDlETXJZOUVaWVI4ZVgxTHV4WVM3RGgxVDNxVHJXTTFGckNVUkxLZVorWnM=

Zhang, J., Liu, J. W., & Wu, Q. L. (2012). A model of goal achievement orientation, self-efficacy, metacognition and academic performance. *Psychological Research, 5(1),* 85-88. Retrieved from http://kns.cnki.net/KCMS/detail/detail.aspx?dbcode=CJFQ&dbname=CJFD2012&filename=OXLY201201016&v=MzA0OTNvOUVZb1I4ZVgxTHV4WVM3RGgxVDNxVHJXTTFGckNVUkxLZVorWnNGeXZrVnI3UEtqWEhkN0c0SDlQTXI=

Zhang, J. X., & Zhang, C. (2008). A comparative study of meta-cognition and academic performance in primary and secondary school students. *Data of Culture and Education, 2008(4),* 95-97. Retrieved from http://kns.cnki.net/KCMS/detail/detail.aspx?dbcode=CJFQ&dbname=CJFDN0508&filename=WJZZ200811048&v=MDAxNTZyV00xRnJDVVJMS2VaK1pzRnlqaFVickpNaWZSZExHNEh0bk5ybzlCYklSOGVYMUx1eFlTN0RoMVQzcVQ=

Zhang, L., & You, Z. Q. (2014). The effects of rural lower secondary school students' belief in a just world, gratitude and time management on their academic achievements. *Chinese Journal of Special Education, 2014(11),* 62-67. Retrieved from http://kns.cnki.net/KCMS/detail/detail.aspx?dbcode=CJFQ&dbname=CJFDLAST2015&filename=ZDTJ201411012&v=MjU2Mjk5WE5ybzlFWm9SOGVYMUx1eFlTN0RoMVQzcVRyV00xRnJDVVJMS2VaK1pzRnlqZ1dyM1BQeW5mWkxHNEg=

Zhang, L. H., Sun, X. J., & Feng, J. Y. (2011). The associations between monitoring, reading performance, and self-regulated learning of elementary school students. *Mental health of Primary and Secondary School, 2011(6),* 10-12. Retrieved from http://kns.cnki.net/KCMS/detail/detail.aspx?dbcode=CFJD&dbname=CJFDN0911&filename=ZXXK201111006&v=MTQxMjR1eFlTN0RoMVQzcVRyV00xRnJDVVJMS2VaK1pzRnlyaFVMekJQelhUWmJHNEg5RE5ybzlGWW9SOGVYMUw=

Zhang, W. (2013). Related research on students' self-efficacy and mathematic achievement in the third grade of junior middle school. *Journal of Nanchang Teaching College, 28(3),* 109-110. Retrieved from http://kns.cnki.net/KCMS/detail/detail.aspx?dbcode=CJFQ&dbname=CJFD2013&filename=LCYY201303070&v=MDU3MjV4WVM3RGgxVDNxVHJXTTFGckNVUkxLZVorWnNGeWpnVjd2SktTN1NkN0c0S

DlMTXJJOUNaSVI4ZVgxTHU=

Zhang, W, H., & Shen, J. L. (2006). A study on the relationship between middle school students' academic stress, achievement goal orientation and academic achievement. *Journal of Southwest China Normal University, 32(6),* 95-98. Retrieved from http://kns.cnki.net/KCMS/detail/detail.aspx?dbcode=CJFQ&dbname=CJFD2006&filename=XBSW200606019&v=MDU2NTBmTXFZOUViWVI4ZVgxTHV4WVM3RGgxVDNxVHJXTTFGckNVUkxLZVorWnNGeWpnVWJ2SVBTL1llYkc0SHQ=

Zhang, X. H., & Gao, C. Y. (2010). A correlational study of gender difference, meta-cognition and English listening performance. *Overseas English, 2010(8),* 339-340. Retrieved from http://kns.cnki.net/KCMS/detail/detail.aspx?dbcode=CJFQ&dbname=CJFD2010&filename=HWYY201008157&v=MjI1OTdXTTFGckNVUkxLZVorWnNGeWpnVTczQUxUclNkN0c0SDlITXA0NUFZNFI4ZVgxTHV4WVM3RGgxVDNxVHI=

Zhang, X. X., & Yang, Y. X. (2012). A correlational study of junior high school students' self-esteem, achievement goal and academic performance. *Education Forum, 2012(10),* 155-156. Retrieved from http://kns.cnki.net/KCMS/detail/detail.aspx?dbcode=CJFU&dbname=CJFDN1214&filename=JTDS201210116&v=MjM2NDViRzRIOVBOcjQ1RVlvUjhlWDFMdXhZUzdEaDFUM3FUcldNMUZyQ1VSTEtlWitac0Z5am5WYnJLTHpuUGY=

Zhang, Y., & Li, Q. W. (2010). The relationships of junior high school students' teacher-student relationships, attribution style and achievement goal orientation with academic achievement. *Psychological Science, 33(4),* 785-788. Retrieved from http://kns.cnki.net/KCMS/detail/detail.aspx?dbcode=CJFQ&dbname=CJFD2010&filename=XLKX201004006&v=MTMyNjBQU0hBZHJHNEg5SE1xNDlGWW9SOGVYMUx1eFlTN0RoMVQzcVRyV00xRnJDVVJMS2VaK1pzRnlqblZML0w=

Zhang, Y. F., Wei, X. C., & He, H. (2014). A comparative study of attribution style and academic performance in senior high school students. *Educational Practice and Research, 2014(11),* 5-9. Retrieved from http://kns.cnki.net/KCMS/detail/detail.aspx?dbcode=CJFQ&dbname=CJFDLAST2015&filename=JYSJ201411003&v=MzI3NTlNTHpUWVpMRzRIOVhOcm85Rlo0UjhlWDFMdXhZUzdEaDFUM3FUcldNMUZyQ1VSTEtlWitac0Z5am5Wcno=

Zhou, L. H., & Liu, Y. (2012). The impact factors of physic academic performance of senior high school students. *The Physical Education Research and Curriculum Theory, 2012(3),* 105-107. Retrieved from http://kns.cnki.net/KCMS/detail/detail.aspx?dbcode=CFJD&dbname=CJFDN1214&filename=WLTB201203041&v=MjY1NTkxRnJDVVJMS2VaK1pzRnlqblU3ckxNaUhmYkxHNEg5UE1ySTlCWllSOGVYMUx1eFlTN0RoMVQzcVRyV00=
